# Intracranial EEG-Based Directed Functional Connectivity in Alpha to Gamma Frequency Range Reflects Local Circuits of the Human Mesiotemporal Network

**DOI:** 10.1007/s10548-024-01084-w

**Published:** 2024-10-22

**Authors:** Yulia Novitskaya, Andreas Schulze-Bonhage, Olivier David, Matthias Dümpelmann

**Affiliations:** 1 Epilepsy Center, Department of Neurosurgery, Faculty of Medicine, University of Freiburg, Breisacher Strasse 64, 79106 Freiburg, Germany; 2 Center for Basics in NeuroModulation, Faculty of Medicine, University of Freiburg, Breisacher Strasse 64, 79106 Freiburg, Germany; 3 Université Grenoble Alpes, Inserm, U1216, Grenoble Institute of Neurosciences, Grenoble, France; 4 Aix Marseille University, Inserm, U1106, INS, Institut de Neurosciences des Systèmes, Marseille, France; 5 Department of Microsystems Engineering (IMTEK), University of Freiburg, Freiburg, Germany

**Keywords:** Directed connectivity, Functional connectivity, Frequency bands, Human temporal lobe, Intracranial EEG

## Abstract

To date, it is largely unknown how frequency range of neural oscillations measured with EEG is related to functional connectivity. To address this question, we investigated frequency-dependent directed functional connectivity among the structures of mesial and anterior temporal network including amygdala, hippocampus, temporal pole and parahippocampal gyrus in the living human brain. Intracranial EEG recording was obtained from 19 consecutive epilepsy patients with normal anterior mesial temporal MR imaging undergoing intracranial presurgical epilepsy diagnostics with multiple depth electrodes. We assessed intratemporal bidirectional functional connectivity using several causality measures such as Granger causality (GC), directed transfer function (DTF) and partial directed coherence (PDC) in a frequency-specific way. In order to verify the obtained results, we compared the spontaneous functional networks with intratemporal effective connectivity evaluated by means of SPES (single pulse electrical stimulation) method. The overlap with the evoked network was found for the functional connectivity assessed by the GC method, most prominent in the higher frequency bands (alpha, beta and low gamma), yet vanishing in the lower frequencies. Functional connectivity assessed by means of DTF and PCD obtained a similar directionality pattern with the exception of connectivity between hippocampus and parahippocampal gyrus which showed opposite directionality of predominant information flow. Whereas previous connectivity studies reported significant divergence between spontaneous and evoked networks, our data show the role of frequency bands for the consistency of functional and evoked intratemporal directed connectivity. This has implications for the suitability of functional connectivity methods in characterizing local brain circuits.

**Supplementary Information:**

The online version contains supplementary material available at 10.1007/s10548-024-01084-w.

## Introduction

Over the past decades, studies of human brain networks received growing attention as the assessment and modelling of connectivity in the brain is a topic of high impact with potential application in the understanding of human brain organization under both physiological as well as various pathological conditions. Human brain connectivity research in vivo is mostly limited to non-invasive brain mapping techniques, such as diffusion-weighted imaging, functional MRI, non-invasive brain stimulation, EEG, and MEG, each one with its own advantages and disadvantages. Under specific in-hospital conditions, intracranial EEG signal in the living human brain can be recorded in patients with therapy resistant epilepsy as a part of presurgical evaluation. Presurgical epilepsy diagnostics is designed to delineate an epileptogenic focus, and due to its very diverse localisation provides wide opportunities for intracranial electrophysiological studies in neuroscience. Intracranial EEG signal is precisely defined in space and time and can be acquired from deep brain structures (Gonzalez-Martinez 2016; Khoo et al. 2020). Similarly to skull EEG, intracranial EEG obtains signal in diverse frequency ranges, extending our knowledge about frequency-specific neural interactions underlying different brain processes, e.g. cognition (Goyal et al., 2020) or changes during the sleep–wake states (Latreille et al., 2020).

There are basically two approaches to assess brain connectivity in the EEG-based signal: evaluation of spontaneous neuronal oscillations during ongoing brain activity (Mayhew et al. 2013; Cantou et al. 2018) and analysis of the electrophysiological neuronal responses, evoked by a SPES, single pulse electrical stimulation (Lacruz et al. 2007; David et al. 2010; Mandonnet et al. 2010; Keller et al. 2014; Kunieda et al. 2015; Krieg et al. 2017).

Analysis of spontaneous neuronal oscillations based on various types of linear (Kramer et al. 2009) and non-linear (Lehnertz 1999; Elger et al. 2000) relationships between the ongoing EEG dynamics recorded in regions of interest allows assessment of *functional connectivity*, which is defined as "temporal correlation of a neurophysiological index measured in different brain areas” (Friston 1994, 2011; Daunizeau et al. 2011). The Granger causality analysis of functional connectivity is one of the most common methods that has been successfully applied in neuroscience in a large number of studies. The method is based on the statistical dependencies between different neural signals estimated through time or frequency domain correlations (Friston 2011; Bastos and Schoffelen 2016), thus providing a quantitative assessment of the directed frequency-specific influences (Chen et al. 2006; Friston et al. 2014; Kaminski et al. 2016; Stokes and Purdon 2017).

Numerous alternative causality measures have been introduced in recent decades, including directed transfer function (DTF) (Kaminski and Blinowska 1991; Kaminski et al. 2016) and partial directed coherence (PDC) (Baccalá and Sameshima 2001; Heyse et al. 2021). Both DTF and PDC are designed on the background of Granger causality and considered to be insensitive to volume conduction as well as tolerant towards noise (Baccalá and Sameshima 2001; Blinowska 2011; Baccalá et al. 2016; Bastos and Schoffelen 2016; Heyse et al. 2021). DTF is an estimator of the intensity of neuronal activity flow between structures in sense of both direct (i.e., the immediate causal influence path) and indirect (the signal traveling through intermediate structures rather than an instant direct causal influence path) directional signal propagation (Baccalá and Sameshima 2001; Baccalá et al. 2016). PDC might be interpreted as an indicator of the level of synchronization between two signals in the studied signal set, because the shared influence from all other signals has been removed. Thus, the PDC might be used as a measure of the strength of direct connection between two structures (Korzeniewska et al. 2003; Heyse et al. 2021).

The SPES approach refers to quantification of the directed causal influence that neuronal population in one brain area exerts over another, so called *effective connectivity* (Friston 1994, 2011). For this purpose, a focal direct intracranial cortical stimulation that evokes electrophysiological responses (corticocortical evoked potentials, CCEP) at the other intracranial recording sites has to be performed. The SPES method has been shown to provide a stable and highly reproducible measure of effective connectivity, however, it requires an active interaction with neuronal circuits (Catenoix et al., 2005; Catenoix et al., 2011; David et al., 2013) Measurements of functional connectivity such as Granger causality, DTF or PDC can be applied on the ongoing neuronal activity, do not require any direct intervention in the nervous system and can be implemented either in the absence of identifiable stimulus or in the context of task performance.

Correlation between functional and effective connectivity still remains unclear. A few previous studies reported rather divergent information (Hebbink et al. 2019; Khastkhodaei et al. 2021; Crocker et al. 2021). Assumingly, different types of neural connectivity are based on the same underlying anatomical networks. Bidirectional SPES-based effective connectivity in the human temporal lobe has been shown to be in exact accord with the anatomical pathways in tracing studies in non-human primates (Novitskaya et al., 2020). Considering neural connectivity as a result of activation of a given neural path, it is rather to expect that both effective connectivity and resting state functional connectivity show similar patterns. In the current study, we compare effective und frequency-dependent functional connections among the structures of anterior and mesial temporal network in the living human brain.

## Methods

### Data Acquisition

**Fig. 1:**
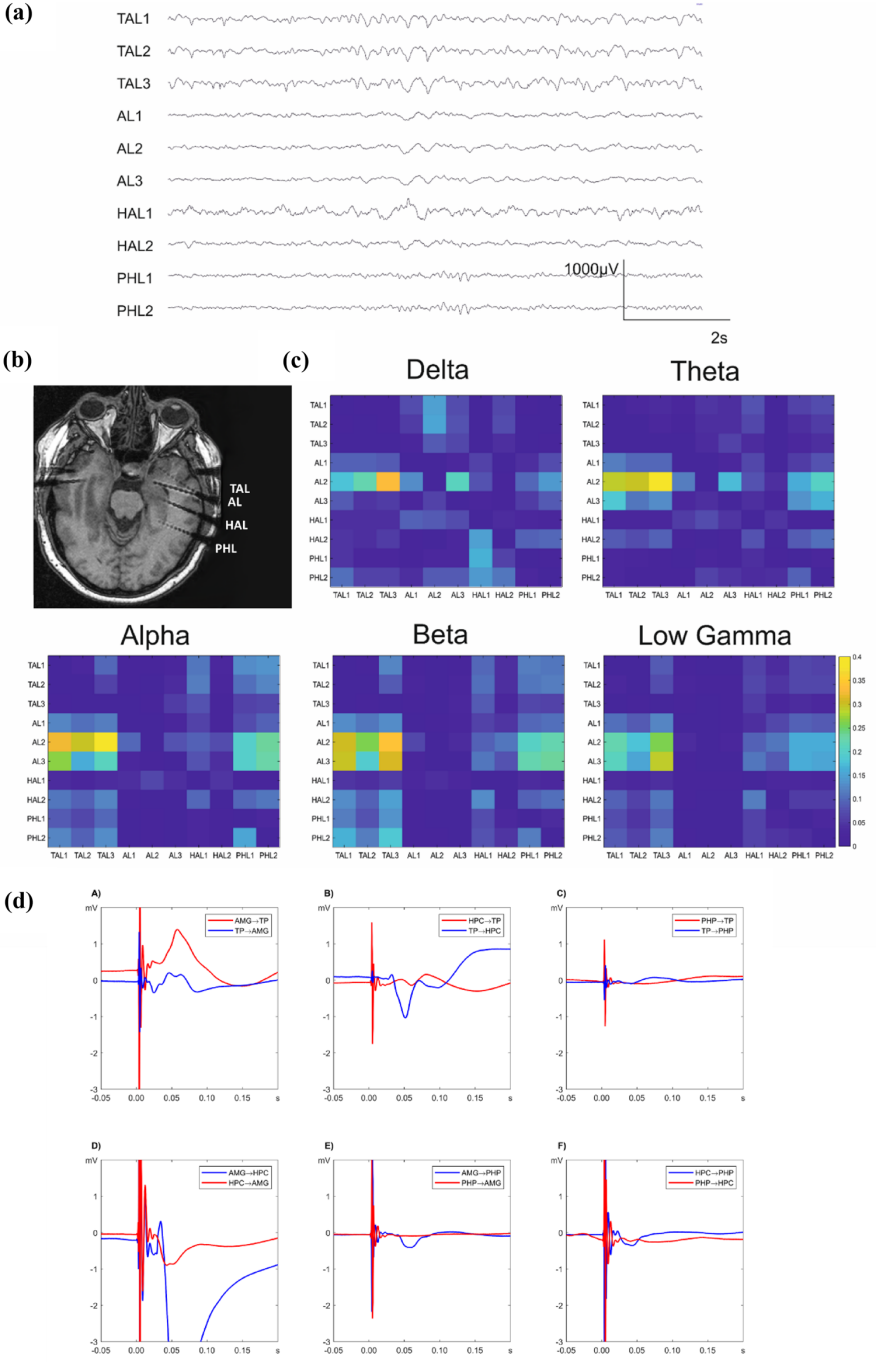
Exemplary data of one representative patient (Pt. 6). **a** An intracranial EEG interictal sample with spontaneous neural activity selected for the Granger Causality analysis. The channel names indicate the electrode placement: *TAL* temporal pole left, *AL* amygdala left, *HAL* hippocampus left, *PHL* parahippocampal gyrus left. The smallest contact number corresponds with the most mesial localization. **b** Postimplantation T1 MRI scan showing a typical placement of the depth electrodes in the left mesial and anterior temporal lobe. **c** Connectivity matrices for the investigated frequency bands based on Granger causality analysis. The source and the target of the Granger causality index are assigned to the y- and x-axis respectively, the matrix labels correspond with the channel names of the depth electrodes. For all frequency bands, high functional connectivity directed from amygdala to the temporal pole and the parahippocampal gyrus is evident, although a decrease in connectivity in slower frequencies is noticeable. **d** Averaged early SPES responses for the six regional pairs arranged according to the source and target of electrical stimulation in a bidirectional way. Asymmetry in bidirectional connectivity is especially noticeable for amygdala and hippocampus (subplot D) as well as hippocampus and temporal pole (subplot B). HPC: hippocampus, *AMG* amygdala, *TP* temporal pole, *PHP* parahippocampal gyrus

**Fig. 2:**
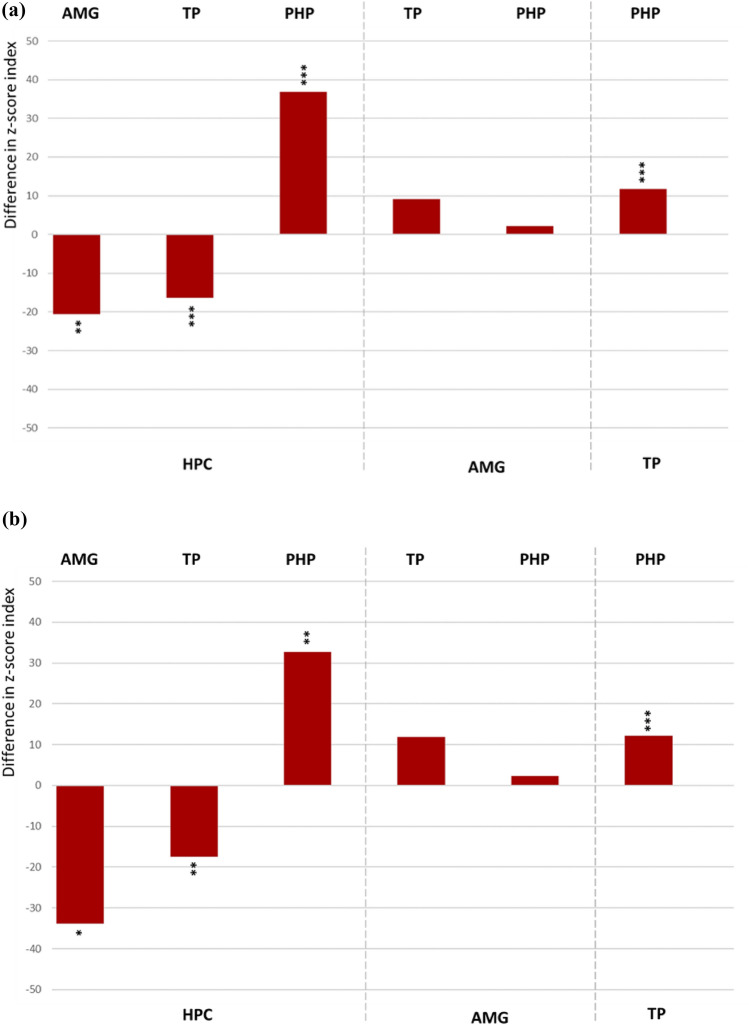
**a** Difference in the z-score index (z-scored measure of amplitude of the evoked responses) demonstrating prevailing directionality in the bidirectional connections among the ROI pairs. The up- or downward orientation of the bars on the zero line indicates the direction of connectivity towards the ROIs assigned above and below the plot. Respectively, the ROIs of signal origin are assigned on the opposite side of the plot. *HPC* hippocampus, *AMG* amygdala, *TP* temporal pole, *PHP* parahippocampal gyrus. ***p < 0.001, **p < 0.01, *p < 0.5. **b** Difference in the z-score index (z-scored measure of amplitude of the evoked responses) demonstrating prevailing directionality in the bidirectional connections among the ROI pairs after exclusion of all contacts in SOZ

Intracranial EEG data, recorded with depth electrodes in nineteen consecutive drug-resistant epilepsy patients (10 males, mean age 36.0 years old, range 16–54 years old) who underwent diagnostical intracranial EEG at the Epilepsy Centre of University Hospital Freiburg in the time period between 2015 and 2019, were used for the study. The study was approved by the Ethics Committee of the University of Freiburg, Germany (reference number 299/14) and written informed consent was obtained from each participant. The inclusion criteria considered (1) intracranial recording fully or partially covering amygdala, hippocampus, temporal pole and parahippocampal gyrus (Fig. 1b), (2) absence of a structural lesion in the above-mentioned structures on 3 T MRI preimplantation scan and (3) participation in a study on single pulse electrical stimulation (SPES). Indication to undergo intracranial EEG and the sites of electrode implantation were based exclusively on clinical reasons in order to localize and delineate the seizure onset zone, therefore the electrodes placement varied between the subjects (subjects’ details are given in Table 1). Standard Montreal Neurological Institute (MNI) coordinates of the contacts placed in the ROIs were identified in each post-implantation 3D T1 image, providing the following averaged MNI coordinates along with the standard deviation (SD) across all participants: amygdala (X: 27.9 (3.7), Y: − 3.0 (2.8), Z: − 20.1 (2.8)), hippocampus (X: 31.6 (3.4), Y: − 12.8 (2.4), Z: − 19.7 (2.6)), parahippocampal gyrus (X: 34.2 (3.8), Y: − 29.9 (7.8), Z: − 17.0 (4.1)), temporal pole (X: 34.1 (5.2), Y: 7.1 (4.8), Z: − 38.1 (3.9)). Seven patients of the study group were implanted in both hemispheres; in total, the data were obtained from 24 implanted hemispheres (15 right) (Table 1).

**Table 1:** Patients profile

Pt	Age/sex	Implanted hemisphere	MRI pathology	SOZ
1	16 M	Right	Nonlesional	Frontal basal R
2	54 F	Right	Encephalocele temporal basal R	Temporal mesial R
3	28 M	Left	Encephalocele temporal basal L	Temporal polar L
4*	39 F	Bilateral	Ganglioglioma WHO I temporal R	Temporal polar R
5	48 M	Bilateral	FCD occipital R	Occipital R
6	54 F	Right	Nonlesional	Non localizable
7*	45 M	Bilateral	HS R, heterotopy frontal L	Temporal polar R and L
8	23 F	Left	Nonlesional	Temporal mesial L
9	38 M	Right	FCD frontal R	Frontal R
10	45 F	Bilateral	Encephalocele temporal basal R and L	Temporal polar R
11	34 M	Right	Nonlesional	Non localizable
12	28 F	Bilateral	Nonlesional	Temporal mesial R
13	35 F	Right	Nonlesional	Temporal mesial L
14	43 F	Bilateral	Encephalocele temporal basal R and L	Temporal polar L
15	26 F	Left	Nonlesional	Temporal mesial L
16	17 M	Right	Nonlesional	Temporal lateral R
17	32 M	Right	Nonlesional	Temporal mesial R
18	45 M	Right	Nonlesional	Temporal mesial and polar R
19	48 M	Bilateral	Nonlesional	Temporal mesial R

The subjects 4 and 7 were implanted bilateral, however, due to a unilateral structural temporal lesion, only data obtained from the nonlesional temporal lobe (in both cases left) were used for the analysis of connectivity

*SOZ* seizure onset zone, *FCD* focal cortical dysplasia, *HS* hippocampal sclerosis

We considered two segments of intracranial EEG data for each participant: ongoing interictal recordings for the analysis of functional connectivity, and SPES data to assess effective connectivity. Both segments were analysed in a referential montage where the reference electrode was placed on the forehead skin and was unaffected by SPES. This approach further allows to assign activity to anatomic regions compared to bipolar channels between adjacent contacts representing local potential differences only. For each proband, the seizure onset zone was defined by two board-certified epileptologists who visually identified the ictal intracranial EEG patterns according to different parameters, including frequency, spatial distribution, and morphology (Gnatkovsky et al., 2019; Lee et al., 2000). In order to eliminate the influence of seizure onset zone on the functional connectivity, the electrode contacts implanted in the seizure onset zone were excluded for corresponding calculation. The data were analyzed and illustrated for both conditions, with and without seizure onset zone, in order to investigate the impact of epileptogencity.

### Evaluation of Functional Connectivity: Pre-processing

For analysis of the functional network, interictal recording of 10 min duration was obtained from awake task-free intracranial EEG starting 30 min before the SPES, sampled at a 2000 Hz sampling rate on a 256-channels DC amplifier (Compumedics, Abbotsford, Australia). Inracranial EEG was submitted to the subsequent analysis chain as recorded against a scalp electrode placed at the forehead. DC contents of the intracranial EEG data was removed using a high-pass filter with a cut-off frequency of 0.5 Hz. To ensure a reasonable model order for estimates of the Granger causality for the classical EEG frequency bands, data were downsampled to a sampling frequency of 250 Hz. The ten minutes interval for the investigation of the Granger causality was subsequently divided into blocks of 10 s. Blocks without movement artefacts and with as less as possible ongoing specific epileptic activity like rhythmical slowing (Fig. 1a) were selected for the model estimation forming the basis for the Granger Causality analysis and derived metrics. Epochs with epileptic activity were excluded first to minimize effects of pathologic synchronization and second, to mitigate a major cause of non-stationarities of the signals. The multivariate models are computed independently for the 10 s segments and the final connectivity matrices were obtained by averaging over the metrics for the single epochs. To assess stability of the resting state connectivity measures the epochs were split into two halves representing early and late epochs. Connectivity measures for both halves are presented in the supplementary material (Sect. 2) and compared against the measures for the complete interval.

### Evaluation of Functional Connectivity: Granger Causality

The Granger causality test is a statistical hypothesis test to determine to which degree one time series can predict another one (Granger 1988). The frequency domain representation of Granger causality metrics can be estimated by a parametric approach using a multivariate autoregressive model (Wen et al. 2013) and from a non-parametric spectral approach using the FFT-algorithm and conjugate multiplication to get cross-spectral densities (Dhamala et al. 2008). In this work, the connectivity metrics were based on the multivariate autoregressive model ensuring connectivity estimates, which are smooth in terms of spectral properties (Bastos and Schoffelen 2016).

The representation of a recording with n signals x_i_(t) in terms of multivariate autoregressive model of order p can be described by the following linear equation:

$$\left[ {\begin{array}{*{20}c} {\begin{array}{*{20}c} {x_{1} \left( t \right)} \\ {x_{2} \left( t \right)} \\ \end{array} } \\ \vdots \\ {x_{n} \left( t \right)} \\ \end{array} } \right] = \mathop \sum \limits_{k = 1}^{p} \left[ {\begin{array}{*{20}c} {\begin{array}{*{20}c} {A_{11} \left( k \right)} & {A_{21} \left( k \right)} \\ {A_{12} \left( k \right)} & {A_{22} \left( k \right)} \\ \end{array} } & \cdots & {\begin{array}{*{20}c} {A_{1n} \left( k \right)} \\ {A_{2n} \left( k \right)} \\ \end{array} } \\ \vdots & \ddots & \vdots \\ {\begin{array}{*{20}c} {A_{n1} \left( k \right)} & {A_{n2} \left( k \right)} \\ \end{array} } & \cdots & {A_{nn} \left( k \right)} \\ \end{array} } \right]\left[ {\begin{array}{*{20}c} {\begin{array}{*{20}c} {x_{1} \left( {t - k} \right)} \\ {x_{2} \left( {t - k} \right)} \\ \end{array} } \\ \vdots \\ {x_{n} \left( {t - k} \right)} \\ \end{array} } \right] + \left[ {\begin{array}{*{20}c} {\begin{array}{*{20}c} {\varepsilon_{1} \left( t \right)} \\ {\varepsilon_{2} \left( t \right)} \\ \end{array} } \\ \vdots \\ {\varepsilon_{n} \left( t \right)} \\ \end{array} } \right],$$

where A_ij_(k) is the coefficient of the autoregressive model at the lag k and ε_i_(t) is a white noise residual with zero mean. Ensemble averaging and transformation to the frequency domain leads to the spectral density matrix:

$$S\left( \omega \right) = H\left( w \right)C_{n} {\text{n}}H^{*} \left( \omega \right),$$

where

$$H\left( \omega \right) = \left( {I - \mathop \sum \limits_{k = 1}^{p} A\left( k \right)e^{ - jk\omega } } \right)^{ - 1} ,$$

and ${C}_{n}$ is the covariance matrix of the noise vector and H(ω) the transfer matrix of the model. Granger causality results in the frequency domain were computed using the BSMART toolbox (freely available for download online under the GNU general public license https://brain-smart.org/). Implementation is based on the publication by (Geweke 1982) and described in (Cui et al. 2008). Model order was selected per patient automatically between 8 and 40 based on the Schwarz's Bayesian Criterion. Granger Causality was determined using the elements of the matrix $H\left(\omega \right)$ with steps of 1 Hz for the classical EEG frequency bands (delta: 1–4 Hz, theta: 5–8 Hz, alpha: 9–12 Hz, beta: 13–24 Hz) and the low gamma band (25 –40 Hz).

### Evaluation of Functional Connectivity: Directed Transfer Function

The Direct Transfer Function (DTF) (Kaminski and Blinowska 1991; Blinowska 2011) normalizes the elements of the transfer matrix by the following formula

$$DTF_{j \to i}^{2} \left( \omega \right) = \frac{{\left| {H_{ij} \left( w \right)} \right|^{2} }}{{\mathop \sum \nolimits_{m = 1}^{k} \left| {H_{im} \left( \omega \right)} \right|^{2} }},$$

where H_*ij*_ is an element of the transfer matrix of the multivariate autoregressive model. Thus, it represents the ratio between the inflow from channel *j* to channel *i* with respect to all the inflows to channel *i*. Values of the DTF are between 0.0 and 1.0.

### Evaluation of Functional Connectivity: Partial Directed Coherence

Partial Directed Coherence normalizes the values of the matrix of the autoregressive model in the frequency domain, producing a ratio between the inflow from channel *j* to channel *i* in respect to all the inflows to channel *i* (Baccalá and Sameshima 2001)

$$PDC_{ij} \left( \omega \right) = \frac{{A_{ij} \left( \omega \right)}}{{\sqrt {a_{j}^{*} \left( \omega \right)a_{j} \left( \omega \right)} }},$$

where A_*ij*_(ω) denotes an element of Fourier transformed MVAR coefficients A(k). The a_*j*_(ω) denotes the j-th column of the matrix A(ω), and an asterisk marks the operation of complex conjugation and transposition. PDC is normalized in the range from 0.0 to 1.0. It represents a ratio between the outflow from channel *j* to channel *i* to all the outflows from the source channel *j*, so it emphasizes rather the sinks, not the sources.

PDC and DTF are estimated using the Biosig Toolbox (freely available for download online under the GNU general public license https://biosig.sourceforge.net/ (Schlögl and Brunner 2008) based on a multivariate autoregressive model as described for the estimate of the Granger causality index.

### Evaluation of Effective Connectivity: Analysis of SPES Network

Each patient participated in a SPES study for connectivity mapping. The SPES procedure was described in details in our previous work (Novitskaya et al. 2020). Briefly, SPES trials were performed in awake patients in task-free interictal phases during the stimulation. Electrical stimulation was applied via two adjoining contacts of the implanted depth electrodes in a bipolar manner at all contact pairs (biphasic square wave pulse of 2 ms duration, 4 s of time interval between the pulses with a jitter of 10%). Current strength (2–4 mA) was adjusted individually for each subject and was limited by either subjective perception of stimulation or the occurrence of evoked epileptic patterns. Forty stimuli per contact pairs were delivered in each session. The recorded stimulus-evoked potentials were cut out of the raw recording off-line limited to 1 s before stimulation and 2 s after stimulation and exported to the Brainstorm software. After baseline correction and averaging according to the time of stimulus onset, the obtained waveforms were reviewed in the Brainstorm display time series panel and processed using a custom designed script for statistical analysis.

Evoked potentials were obtained from all non-stimulating intracranial electrodes that were detected in the grey matter of the regions of interest. The SPES network was constructed based on early CCEP-components (N1-components, Matsumoto et al. 2007; Keller et al. 2014) that were analysed by calculating z-score of the maximum signal amplitude in the 100-ms time window starting 10 ms after the stimulus application, in order to exclude the stimulation artefacts (Donos et al. 2016). Each response to stimulation represented a specific combination of a stimulation and a recording contact in the regions of interest of temporal network. Therefore, all obtained evoked responses were arranged in six stimulation-response groups depending on the origin of the stimulus and the point of the response recording. The quantification of the CCEPs in a z-score value allowed a direct comparison of the different stimulation-response groups.

### Statistics

The data were evaluated by performing a univariate analysis of variance (ANOVA) on incoming and outgoing measurements of the GC, PDC and DTF index as well as z-scored CCEP amplitudes between all electrode pairs within the target structures, followed by Bonferroni post-hoc test when appropriate. The regional pairs, i.e. origin of the signal and targeted endpoint, were used as a between-subject (group) factor. To proof the existence of a predominant direction the differences between the elements i, j and j, i of the of the connectivity matrices was determined. The null hypothesis of the presence of a zero mean distribution for these differences was tested by a one sample t-test with zero as the assumed mean. Results of these tests are presented in the supplementary material (Sect. 1). The SPSS Statistics (v.22) software package was used for statistical analysis. Results were considered significant when p < 0.05.

## Results

In total, the analysed intracranial EEG segments were obtained from 266 recording sites in amygdala, 267—in hippocampus, 235—in parahippocampal gyrus, and 308—in the temporal pole. For connectivity analysis, the selected regions were arranged in six bidirectionally connected source-target groups, where the source was the region of signal origin and the target was the recording area of resultant spontaneous activity for analysis of functional connectivity. For the analysis of effective connectivity, the source was the area of electrical stimulation and the target was the recording area of evoked responses. For the multivariate autoregressive models being the basis of analysis of functional connectivity model orders between 8 and 40 with a median value of 15 were determined by the Schwarz's Bayesian Criterion.

Figure 1c demonstrates exemplary functional connectivity matrices based on Granger causality analysis showing unequal strength of connectivity among the ROIs, when compared bidirectionally, especially in the higher frequency bands. Similar for effective connectivity, the SPES evoked responses were found to differentiate in amplitude depending on the source of stimulation (Fig. 1d) that we described in details in our previous work (Novitskaya et al. 2020). These findings formed the basis of the following analysis that resulted in the data presented below.

### Directed Evoked (Effective) Connectivity: SPES Study

The distribution of effective directionality is presented in the Fig. 2a indicating significantly stronger input from amygdala and temporal pole towards the hippocampus (AMG → HPC: F_1,84_ = 7.7, p < 0.01; TP → HPC: F_1,109_ = 18.0, p < 0.001). The parahippocampal incoming connections from hippocampus and temporal pole were significantly stronger than contrariwise (HPC → PHP: F_1,68_ = 15.3, p < 0.001; TP → PHP: F_1,78_ = 46.4, p < 0.001). The amygdalar outgoing connections towards temporal pole and parahippocampal gyrus showed a mild non-significant trend towards asymmetry (AMG → TP: F_1,109_ = 2,8, n.s.; AMG → PHP: F_1,71_ = 0.8, n.s.). Exclusion of recording contacts placed in seizure onset zone did not influence the pattern of intratemporal effective connectivity (Fig. 2b).

### Directed Functional Connectivity Based on Granger Causality

**Fig. 3:**
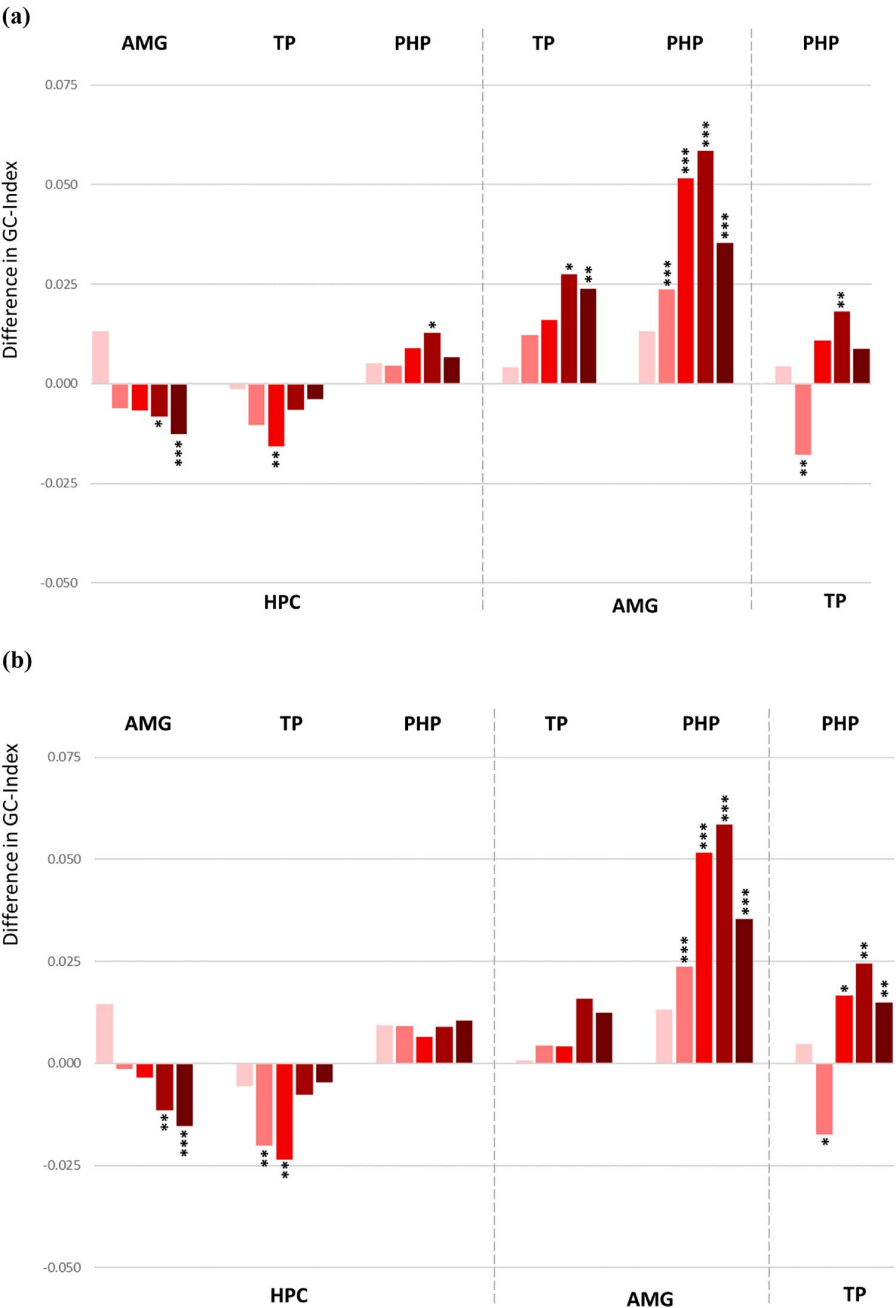
**a** Difference in the Granger Causality index demonstrating prevailing directionality in the bidirectional connections among the ROI pairs. Each bunch of bars represents five colour coded frequency bands: delta (pink), theta (light red), alpha (red), beta (dark red) and low gamma (brown), ordered from left to right. The up- or downward orientation of the bars on the zero line indicates the direction of connectivity towards the ROIs assigned above and below the plot. Respectively, the ROIs of signal origin are assigned on the opposite side of the plot. *HPC* hippocampus, *AMG* amygdala, *TP* temporal pole, *PHP* parahippocampal gyrus. ***p < 0.001, **p < 0.01, *p < 0.5. **b** Difference in the Granger Causality index demonstrating prevailing directionality in the bidirectional connections among the ROI pairs after exclusion of all contacts in SOZ

For statistical evaluation, univariate ANOVA was applied to the resulting Granger causality index in order to explore the causality of bidirectional activation within the mesial temporal network in awake state. The obtained connectivity pattern displayed prominently asymmetrical connections among the ROIs and shared substantial similarities to the CCEP network (Fig. 3a). The statistical details are given in the Supplementary Table 2a. For most of the cases, functional connectivity shows clear directionality when calculated for higher frequency bands as beta or low gamma. In lower frequency bands i.e. delta and theta, progressively less asymmetrical bidirectional connections among the ROIs were revealed, except the connectivity between temporal pole and parahippocampal gyrus that showed an opposite direction in the theta frequency band.

### Directed Functional Connectivity Based on Partial Directed Coherence

**Fig. 4:**
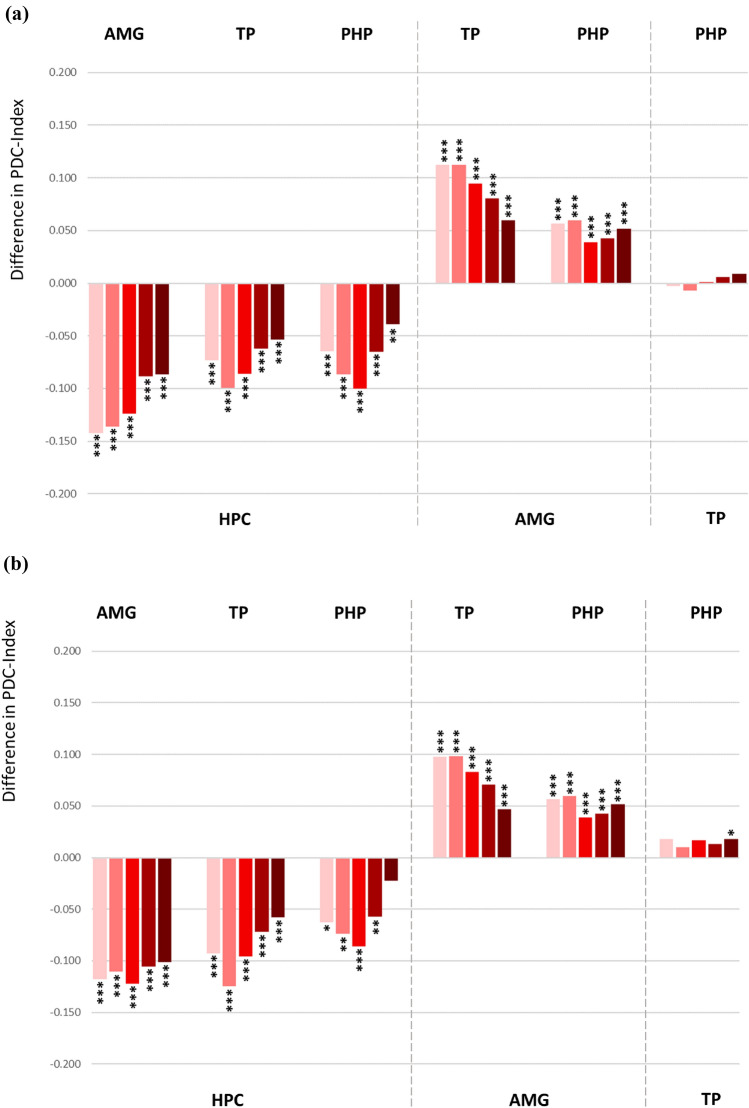
**a** Difference in the PDC (Partial Directed Coherence) index demonstrating prevailing directionality in the bidirectional connections among the ROI pairs. Each bunch of bars represents five colour coded frequency bands: delta (pink), theta (light red), alpha (red), beta (dark red) and low gamma (brown), ordered from left to right. The up- or downward orientation of the bars on the zero line indicates the direction of connectivity towards the ROIs assigned above and below the plot. Respectively, the ROIs of signal origin are assigned on the opposite side of the plot. *HPC* hippocampus, *AMG* amygdala, *TP* temporal pole, *PHP* parahippocampal gyrus. ***p < 0.001, **p < 0.01, *p < 0.5. **b** Difference in the PDC index demonstrating prevailing directionality in the bidirectional connections among the ROI pairs after exclusion of all contacts in SOZ

The Partial Directed Coherence (PDC) method also revealed directionalities in the connections among the ROIs, mostly overlapping with those based on the Granger Causality analysis as described above. The obtained PDC results showed a stable spread among all frequency bands (Fig. 4a). The data indicate statistically stronger outgoing connections from amygdala, temporal pole and parahippocampal gyrus directed towards hippocampus (the statistical details are given in the Supplementary Table 2a). The outgoing connection from amygdala directed to temporal pole and parahippocampal gyrus appeared to be stronger than contrariwise. No significant asymmetry in directionality was found the connections between temporal pole and parahippocampal gyrus.

### Directed Functional Connectivity Based on Directed Transfer Function

**Fig. 5:**
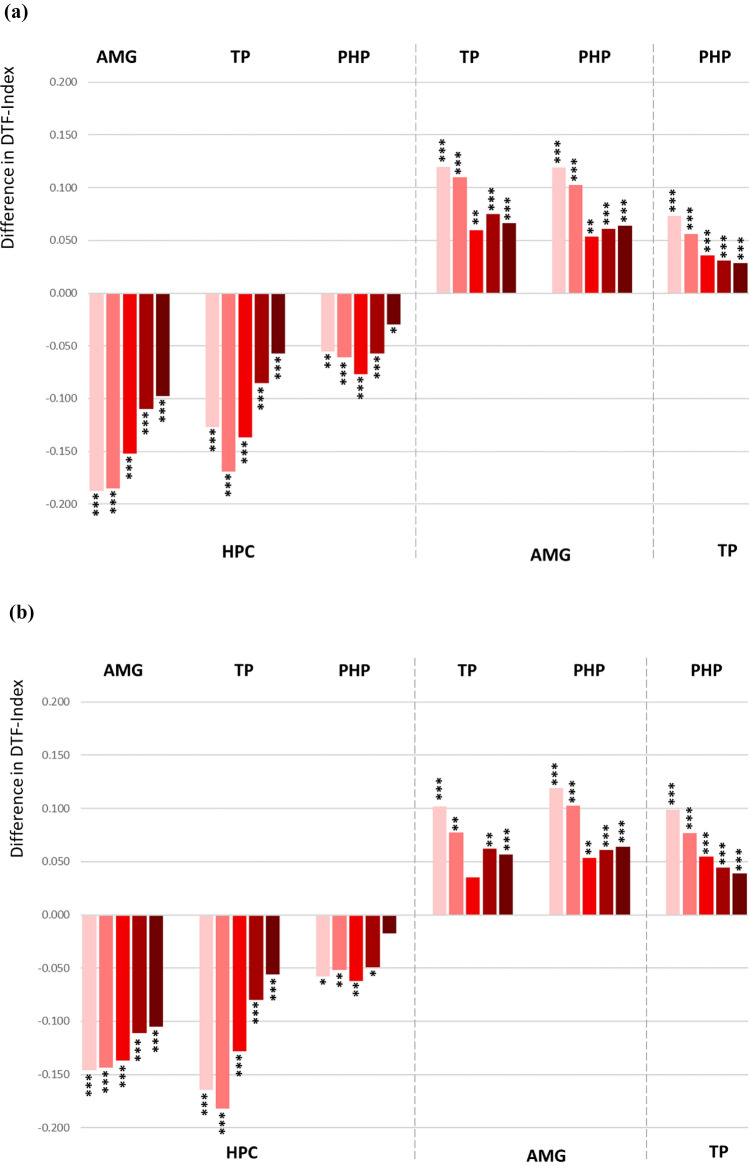
**a** Difference in the DTF (Directed Transfer Function) index demonstrating prevailing directionality in the bidirectional connections among the ROI pairs. Each bunch of bars represents five colour coded frequency bands: delta (pink), theta (light red), alpha (red), beta (dark red) and low gamma (brown), ordered from left to right. The up- or downward orientation of the bars on the zero line indicates the direction of connectivity towards the ROIs assigned above and below the plot. Respectively, the ROIs of signal origin are assigned on the opposite side of the plot. *HPC* hippocampus, *AMG* amygdala, *TP* temporal pole, *PHP* parahippocampal gyrus. ***p < 0.001, **p < 0.01, *p < 0.5. **b** Difference in the DTF index demonstrating prevailing directionality in the bidirectional connections among the ROI pairs after exclusion of all contacts in SOZ

The data analysed by Directed Transfer Function (DTF) method tended to show similar distribution in the directionality as in the PDC analysis, however, the connection between temporal pole and parahippocampal gyrus reached the significant difference indicating stronger efferent modulation from temporal pole towards parahippocampal gyrus (Fig. 5a, Supplementary Table 2a for statistical details). Similar to the PDC method, significant difference was observed in all frequency bands (Fig. 5a, Supplementary Table 2a).

### Functional Connectivity and Seizure Onset Zone

In order to eliminate the effect of seizure onset zone on functional connectivity, we excluded all source and target recording contacts placed in the seizure onset regions that were defined by two intracranial EEG experienced clinicians. By that, the number of analysed intracranial EEG segments was reduced to 216 recording sites in amygdala, 151—in hippocampus, 188—in parahippocampal gyrus, and 229—in the temporal pole.

The exclusion of seizure onset zone did not change the directed connectivity based on Granger Causality method, although, statistical significance was no longer reached for connections between hippocampus and parahippocampal gyrus as well as amygdala and temporal pole (Fig. 3b, the statistical values are given in the Supplementary Table 2b). The efferent modulation from amygdala and temporal pole remained to be strongly directed towards the hippocampus. The amygdalo-parahippocampal connectivity showed prevailed directionality towards the parahippocampal gyrus in almost all frequency bands. Bidirectional functional connectivity between the temporal pole and parahippocampal gyrus also showed significant asymmetry with the stronger input directed from temporal pole towards parahippocampal gyrus in higher frequency bands and in the opposite direction in the theta frequency band.

Directed functional connectivity based on the PDC analysis revealed similar distribution of the intratemporal connections (Fig. 4b) with the exception of connectivity between parahippocampal gyrus and temporal pole, that showed a stronger temporal polar input towards parahippocampal gyrus reaching statistical significance in the low gamma frequency band. The results obtained for the DTF method were identical for the data sets including or excluding the seizure onset zone (Fig. 5b). The statistical details for the PDC und DTF analysis are given in the Supplementary Table 2b.

## Discussion

We investigated frequency-based functional connectivity among structures of the human anterior mesial temporal lobe in vivo, based on analysis of spontaneous neuronal activity recorded in a cohort of epilepsy patients during invasive presurgical monitoring. The obtained connectivity patterns were compared with evoked (effective) connectivity in the same network. Both approaches revealed significant asymmetrical directionality in the strength of feedforward and feedback connections in the anterior mesial temporal network. Intratemporal reciprocal high-frequency (alpha to low gamma) connectivity pattern derived from the Granger causality analysis shared substantial similarities with the patterns found in the evoked network. Among all measurement methods, significantly stronger efferent modulation from amygdala and temporal pole towards hippocampus was found. Directional connectivity among other ROI pairs partially overlapped between the selected methods.

### Evoked (Effective) Connectivity in the Mesiotemporal Network

Early sharp potentials evoked by electrical stimulation in remote brain areas within the first 100 ms after stimulation are considered to be physiological responses proper for mapping cortical connectivity (Valentín et al. 2005a, b). Their generation has been shown to be steady in the presence of epileptic activity (Wilson et al. 1990), however, some studies reported changes in morphology, amplitude and latency of evoked potentials in epileptogenic brain regions (Rosenberg et al. 2009; Kundu et al. 2020; Hays et al. 2021). In our own data, exclusion of electrode contacts placed in the seizure onset zone resulted in similar bidirectional connectivity, suggesting that the established connectivity was not altered to a major degree by the epileptogenicity in the targeted regions. Our data revealed asymmetrically weighed reciprocal intratemporal effective connectivity in the living human brain, accurately corresponding to the available data obtained from anatomical studies in non-human primates, or, when those are missing, in rodents (Witter and Amaral 1991; Stefanacci et al. 1996; Pitkänen et al. 2000; Munoz et al. 2005; Novitskaya et al. 2020). The agreement with primate anatomical studies strongly suggests that the invasive method of direct electrical stimulation provides valid information about effective connectivity in humans in vivo (Seguin et al. 2023).

### Frequency Dependent Functional Connectivity Based on Granger Causality Method

Intratemporal reciprocal connectivity pattern derived from the Granger causality analysis shared substantial similarities to the evoked network revealing stronger amygdala and temporal pole efferents towards hippocampus as well as more pronounced parahippocampal afferents from hippocampus and temporal pole (Fig. 3a). The amygdala input towards temporal pole and parahippocampal gyrus was highly significant in the functional network whereas the connectivity in the SPES network showed basically the same directionality, however, not reaching statistical significance (Fig. 2a).

The Granger causality analysis was designed to provide a quantitative report of the directed frequency-specific influences (Chen et al. 2006; Friston et al. 2014; Kaminski et al. 2016; Stokes and Purdon 2017). Our data suggest that the most prominent directionality of the functional connections was present in beta and low gamma frequency bands with gradual flattening of the directionality along with lower EEG frequencies. The role of frequency bands in the EEG-based functional connectivity assessment has not been systematically investigated yet. Previous studies consider that synchronization in the lower frequencies may support connectivity between physically more distant regions, whereas the higher frequencies are considered to reflect local interactions in the brain (Jones et al. 2000; Kopell et al. 2000; Wu et al. 2008; Samogin et al. 2020).

Our data support the assumption that a higher degree of synchronisation between two closely spaced structures, which underlies the signal directionality, can be observed in higher frequencies such as alpha, beta and low gamma. A lower degree of directionality for slower oscillations may be due to a less fine-grained spatial organization of processes underlying their generation. Depending on their frequency, oscillatory activity has different mechanisms of generation. This includes different rhythm generators (pacemakers) such as subcortical nuclei and thalamus for delta activity (Steriade et al. 1993; Maquet et al. 1997; Amarillo et al. 2018), medial septum and diagonal band of Broca area for theta (Buzsáki 2002; Kocsis et al. 2022), layers III, IV and V of neocortex for alpha (Steriade et al. 1990; Bollimunta et al. 2008; Halgren et al. 2019) as well as local circuits with pyramidal cells for beta (Kopell et al. 2000; Engel and Fries 2010) and GABA-mediated inhibitory interneurons for gamma (Bartos et al. 2007; Susin and Destexhe 2021). In terms of functional connectivity, the wave length of the examined oscillations also reflects the size of the assessed synchronized network. Whereas slow oscillations can recruit many neurons in extended brain areas, fast oscillations reflect more local circuits partwise related to limitations of axon conduction delays. We conclude that functional connectivity of adjacent structures and local modules as analysed here is particularly reflected in the higher frequency bands.

In this work, we assessed early CCEPs in the time window of 100 ms. Considering correlation between effective (CCEP-based) and functional networks, oscillations in higher frequencies are also better comparable with the CCEPs because the higher frequency bands, i.e., alpha and above, have period of oscillations within 100 ms. The lower frequencies, theta and delta, in contrast, do not reach their peaks within 100 ms and, therefore, are less congruent with CCEPs.

The same way as the evoked connectivity, the functional network based on the assessment of Granger Causality did not change significantly depending on the presence or absence of seizure onset zone. Exclusion of all electrode contacts placed in the seizure onset zone resulted in consonant bidirectional functional connectivity (Fig. 3b), suggesting that in this sample of nonlesional epilepsy patients the established connectivity was not altered by epileptogenicity of the assessed target regions.

### Functional Network Based on DTF and PDC

Additionally, we determined the direction and strength of intratemporal functional connectivity using the DTF and PCD methods. The results mainly confirmed the directionality patterns obtained from the assessment based on the Granger causality method, however, with the exception of connectivity between hippocampus and parahippocampal gyrus that appeared to be directed towards hippocampus. Our data did not show frequency-dependent changes in the DTF and PDC based connectivity as a consistent significant bidirectional asymmetry was revealed in all explored frequency bands.

Overall, only interactions between hippocampus and parahippocampal gyrus were differentially characterized when using the different methods applied here. In the SPES study, hippocampal stimulation evoked the largest discharges in the parahippocampal gyrus, clearly indicating asymmetrical reciprocal connections with significant prevalence of the parahippocampal responses (s. Novitskaya et al. 2020 for more details), also confirming the observation in an earlier human SPES study (Enatsu et al. 2015).

Most of the evidence on hippocampal connections comes from investigations in animals stating bilateral connectivity between the hippocampus and parahippocampal gyrus over the entorhinal cortex, “the gateway to hippocampus”. Numerous anatomical tracing studies in monkeys showed that the parahippocampal cortex feeds information into the entorhinal cortex and projects largely to the medial entorhinal area, which in turn projects to the hippocampal formation, providing the heaviest input to the dorsal dentate gyrus and dorsal field CA1 (Insausti and Amaral 2008; Aggleton 2012; Nilssen et al. 2019). The backword hippocampal projections also have been shown in monkeys demonstrating that certain parahippocampal areas receive substantial projections from the CA1 field (Yukie 2000). A recent diffusion tractography study in humans revealed more complicated and widespread hippocampal connections than in monkeys, including reciprocal connections with the parahippocampal gyrus (Huang et al. 2021). The finding suggests underlying mechanisms of informational recall back to the neocortex, since hippocampal formation and the surrounding cortices of the parahippocampal region in humans are known to contribute crucially to learning and declarative memory (Eichenbaum et al. 2007; Ranganath 2010; Rolls et al. 2022). Human fMRI studies suggest that connectivity between hippocampus and parahippocampal gyrus appears to be rather context dependent than static (Wang et al. 2016; Ward et al. 2014; Qin et al. 2016). Thus, a significant task activation has been shown for hippocampus, whereas parahippocampal gyrus was more active during the rest (Ward et al. 2014).

Regarding the impact of epileptiform activity, the DTF based functional network has not been affected by epileptogenicity (Fig. 5b). In the PDC network, most connections were not affected by epileptiform activity either, however, the connectivity between temporal pole and parahippocampal gyrus did change the directionality when the recording contacts placed in seizure onset zone had been excluded from the analysis (Fig. 4b). Showing initially no asymmetry in the connectivity, a significant directionality towards parahippocampal gyrus became apparent in the non-epileptogenic network. Epileptogenicity is associated with abnormal interactions and causal relationships with the surrounding neuronal populations (Sabesan et al. 2009; Wang et al. 2017). The interictal suppression hypothesis suggests that epileptogenic zones have increased inward connectivity which could relate to interictal suppression of epileptiform activity as a control mechanism during seizure-free periods (Vlachos et al. 2017; Narasimhan et al. 2020; Johnson et al. 2023).

In our cohort of epilepsy patients, an epileptogenic focus in the temporal pole was detected in six probands, whereas seizure onset in the parahippocampal gyrus was much less present and was registered merely in three patients in one seizure in every case. Taking into account the interictal suppression hypothesis, our data might suggest a high interictal input towards the epileptogenic temporal pole that was eliminated with the removal of SOZ-placed electrodes, uncovering its efferent influence.

## Conclusion

**Fig. 6:**
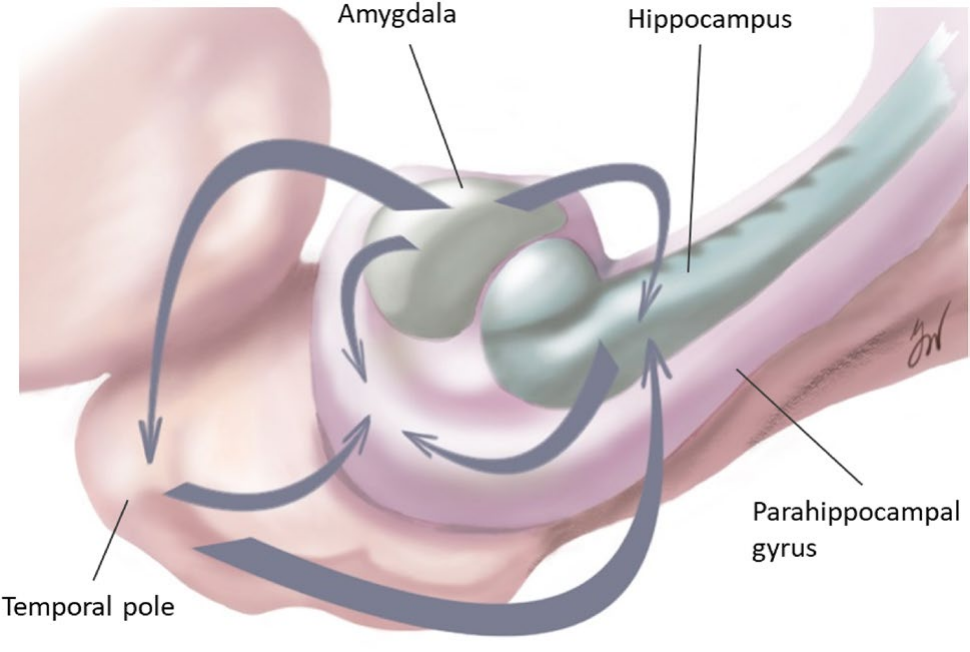
Summary of directed connections within the human mesial temporal lobe, as revealed by the Granger causality analysis of functional connectivity as well as the analysis of efficient connectivity in the SPES network

We assessed frequency-based directed functional connectivity among the structures of the anterior mesial temporal lobe in the living human brain und compared the results with effective connectivity evaluated by means of SPES method. The closest overlapping to the evoked network was found for the functional connectivity assessed by the Granger causality method (Fig. 6). Functional connectivity assessed by means of DTF and PCD obtained basically a similar directionality pattern with the exception of connectivity between hippocampus and parahippocampal gyrus that appeared to be directed contrariwise.

The functional connectivity has been found to overlap with the effective connectivity when assessed in higher frequency bands, possibly as a correlate of synchronized gamma oscillations reflecting coordinated activity in small networks. Using short range connections can be here of advantage since non-evoked connectivity methods, defined as statistical dependencies among remote neurophysiological events, might get overpowered by activity of local circuits when applied to long range connections.

Besides the network size, a further methodological issue might be important. Despite its widespread use, Granger causality and alternative causality estimators remain a matter of debate in neuroscience. The main concern is that measures can be problematic to interpret, as they refer to a combination of different system components those contributions cannot be segregated by examining the causality values alone (David et al. 2008; Bastos and Schoffelen 2016; Stokes and Purdon 2017). Already the selection of the reference or montage is not completely solved. Here it is opted for a referential montage though being susceptible to volume conduction and to comparable high noise contaminations. But, one of the applied measures (DFT) was shown to be not influenced by volume conduction and that using application of a Laplacian or bipolar montage operator ﻿destroys the original correlation structure of the set of signals (Kaminski et al. 2014). Also, divergence between effective and functional networks in vivo was admitted in several recent works (Hebbink et al. 2019; Khastkhodaei et al. 2021; Crocker et al. 2021) reflecting that unlike the evoked connectivity that revealed to be stable under different conditions, functional connectivity can vary state-dependently. Our data, however, suggest that both functional and effective methods are suitable for detection of local circuits, however, application of several measures may be necessarily for a full characterization of the network properties. Temporal stability inside of the interval chosen for resting state analysis could be shown (see supplementary data, Sect. 2) but the inclusion of further intervals e.g. at a different time of day could be an interesting topic for further research. Overall, the data reported here for a physiological resting network provides a background for future studies addressing task-dependent changes in functional connectivity.

## Supplementary Information

Below is the link to the electronic supplementary material.

Supplementary file1 (DOCX 571 kb)

Supplementary file2 (DOCX 28 kb)

## Abbreviations

AMG: Amygdala
CCEP: Corticocortical evoked potentials
DTF: Directed transfer function
EEG: Electroencephalography
FCD: Focal cortical dysplasia
GC: Granger causality
HPC: Hippocampus
HS: Hippocampal sclerosis
MEG: Magnetoencephalography
MVAR: Multivariate autoregressive model
PDC: Partial directed coherence
PHP: Parahippocampal gyrus
SOZ: Seizure onset zone
SPES: Single pulse electrical stimulation
TP: Temporal pole
