# Supplementary material for: Intracranial EEG-Based Directed Functional Connectivity in Alpha to Gamma Frequency Range Reflects Local Circuits of the Human Mesiotemporal Network
Source: Brain Topogr. 2024 Oct 22;38(1):10. doi: 10.1007/s10548-024-01084-w (PMC11496326; doi:10.1007/s10548-024-01084-w)
Supplement: Supplementary file 1 — Supplementary file1 (DOCX 571 kb) [file 10548_2024_1084_MOESM1_ESM.docx]

Supplementary materials.

1. Test for null hypothesis for the predominant direction of connectivity scores of a zero mean distribution. The test was performed by a one tailed t-test with 0 as the reference value (Haufe, S. et al. ‘A critical assessment of connectivity measures for EEG data: A simulation study’, *NeuroImage*, 2013).

Group 1: temporal pole 🡪 amygdala

Group 2: amygdala 🡪 temporal pole

Group 3: temporal pole 🡪 hippocampus

Group 4: hippocampus 🡪 temporal pole

Group 5: temporal pole 🡪 parahippocampal gyrus

Group 6: parahippocampal gyrus 🡪 temporal pole

Group 7: amygdala 🡪 hippocampus

Group 8: hippocampus 🡪 amygdala

Group 9: amygdala 🡪 parahippocampal gyrus

Group 10: parahippocampal gyrus 🡪 amygdala

Group 11: hippocampus 🡪 parahippocampal gyrus

Group 12: parahippocampal gyrus 🡪 hippocampus

**Partial directed coherence**

| **Variable** | **N** | **mean** | **Std. Deviation** | **Std. errorr mean** | **t** | **df** | **Sign.** |
| --- | --- | --- | --- | --- | --- | --- | --- |
| **Group 1** |  |  |  |  |  |  |  |
| PDC Delta | 115 | -0,1096 | 0,14677 | 0,01369 | -8,006 | 114 | 0,000 |
| PDC Theta | 115 | -0,1040 | 0,15878 | 0,01481 | -7,025 | 114 | 0,000 |
| PDC Alpha | 115 | -0,0864 | 0,14955 | 0,01395 | -6,194 | 114 | 0,000 |
| PDC Beta | 115 | -0,0744 | 0,13705 | 0,01278 | -5,819 | 114 | 0,000 |
| PDC Gamma | 115 | -0,0559 | 0,12300 | 0,01147 | -4,877 | 114 | 0,000 |
| **Group 2** |  |  |  |  |  |  |  |
| PDCDelta | 115 | 0,1096 | 0,14677 | 0,01369 | 8,006 | 114 | 0,000 |
| PDCTheta | 115 | 0,1040 | 0,15878 | 0,01481 | 7,025 | 114 | 0,000 |
| PDCAlpha | 115 | 0,0864 | 0,14955 | 0,01395 | 6,194 | 114 | 0,000 |
| PDCBeta | 115 | 0,0744 | 0,13705 | 0,01278 | 5,819 | 114 | 0,000 |
| PDCGamma | 115 | 0,0559 | 0,12300 | 0,01147 | 4,877 | 114 | 0,000 |
| **Group 3** |  |  |  |  |  |  |  |
| PDCDelta | 113 | 0,0720 | 0,14591 | 0,01373 | 5,248 | 112 | 0,000 |
| PDCTheta | 113 | 0,0993 | 0,13801 | 0,01298 | 7,651 | 112 | 0,000 |
| PDCAlpha | 113 | 0,0857 | 0,12619 | 0,01187 | 7,218 | 112 | 0,000 |
| PDCBeta | 113 | 0,0621 | 0,08844 | 0,00832 | 7,459 | 112 | 0,000 |
| PDCGamma | 113 | 0,0516 | 0,07630 | 0,00718 | 7,184 | 112 | 0,000 |
| **Group 4** |  |  |  |  |  |  |  |
| PDCDelta | 113 | -0,0720 | 0,14591 | 0,01373 | -5,248 | 112 | 0,000 |
| PDCTheta | 113 | -0,0993 | 0,13801 | 0,01298 | -7,651 | 112 | 0,000 |
| PDCAlpha | 113 | -0,0857 | 0,12619 | 0,01187 | -7,218 | 112 | 0,000 |
| PDCBeta | 113 | -0,0621 | 0,08844 | 0,00832 | -7,459 | 112 | 0,000 |
| PDCGamma | 113 | -0,0516 | 0,07630 | 0,00718 | -7,184 | 112 | 0,000 |
| **Group 5** |  |  |  |  |  |  |  |
| PDCDelta | 92 | -0,0051 | 0,10427 | 0,01087 | -0,467 | 91 | 0,642 |
| PDCTheta | 92 | -0,0097 | 0,07886 | 0,00822 | -1,183 | 91 | 0,240 |
| PDCAlpha | 92 | -0,0024 | 0,07687 | 0,00801 | -0,299 | 91 | 0,766 |
| PDCBeta | 92 | 0,0026 | 0,06413 | 0,00669 | 0,395 | 91 | 0,694 |
| PDCGamma | 92 | 0,0059 | 0,06490 | 0,00677 | 0,876 | 91 | 0,383 |
| **Group 6** |  |  |  |  |  |  |  |
| PDCDelta | 92 | 0,0051 | 0,10427 | 0,01087 | 0,467 | 91 | 0,642 |
| PDCTheta | 92 | 0,0097 | 0,07886 | 0,00822 | 1,183 | 91 | 0,240 |
| PDCAlpha | 92 | 0,0024 | 0,07687 | 0,00801 | 0,299 | 91 | 0,766 |
| PDCBeta | 92 | -0,0026 | 0,06413 | 0,00669 | -0,395 | 91 | 0,694 |
| PDCGamma | 92 | -0,0059 | 0,06490 | 0,00677 | -0,876 | 91 | 0,383 |
| **Group 7** |  |  |  |  |  |  |  |
| PDCDelta | 92 | 0,1466 | 0,21060 | 0,02196 | 6,678 | 91 | 0,000 |
| PDCTheta | 92 | 0,1375 | 0,20187 | 0,02105 | 6,531 | 91 | 0,000 |
| PDCAlpha | 92 | 0,1216 | 0,20307 | 0,02117 | 5,745 | 91 | 0,000 |
| PDCBeta | 92 | 0,0865 | 0,17761 | 0,01852 | 4,671 | 91 | 0,000 |
| PDCGamma | 92 | 0,0859 | 0,12748 | 0,01329 | 6,463 | 91 | 0,000 |
| **Group 8** |  |  |  |  |  |  |  |
| PDCDelta | 92 | -0,1466 | 0,21060 | 0,02196 | -6,678 | 91 | 0,000 |
| PDCTheta | 92 | -0,1375 | 0,20187 | 0,02105 | -6,531 | 91 | 0,000 |
| PDCAlpha | 92 | -0,1216 | 0,20307 | 0,02117 | -5,745 | 91 | 0,000 |
| PDCBeta | 92 | -0,0865 | 0,17761 | 0,01852 | -4,671 | 91 | 0,000 |
| PDCGamma | 92 | -0,0859 | 0,12748 | 0,01329 | -6,463 | 91 | 0,000 |
| **Group 9** |  |  |  |  |  |  |  |
| PDCDelta | 78 | 0,0549 | 0,13354 | 0,01512 | 3,628 | 77 | 0,001 |
| PDCTheta | 78 | 0,0579 | 0,08915 | 0,01009 | 5,740 | 77 | 0,000 |
| PDCAlpha | 78 | 0,0390 | 0,07278 | 0,00824 | 4,738 | 77 | 0,000 |
| PDCBeta | 78 | 0,0421 | 0,07225 | 0,00818 | 5,147 | 77 | 0,000 |
| PDCGamma | 78 | 0,0499 | 0,07891 | 0,00893 | 5,585 | 77 | 0,000 |
| **Group 10** |  |  |  |  |  |  |  |
| PDCDelta | 78 | -0,0549 | 0,13354 | 0,01512 | -3,628 | 77 | 0,001 |
| PDCTheta | 78 | -0,0579 | 0,08915 | 0,01009 | -5,740 | 77 | 0,000 |
| PDCAlpha | 78 | -0,0390 | 0,07278 | 0,00824 | -4,738 | 77 | 0,000 |
| PDCBeta | 78 | -0,0421 | 0,07225 | 0,00818 | -5,147 | 77 | 0,000 |
| PDCGamma | 78 | -0,0499 | 0,07891 | 0,00893 | -5,585 | 77 | 0,000 |
| **Group 11** |  |  |  |  |  |  |  |
| PDCDelta | 76 | -0,0647 | 0,16529 | 0,01896 | -3,415 | 75 | 0,001 |
| PDCTheta | 76 | -0,0894 | 0,15733 | 0,01805 | -4,955 | 75 | 0,000 |
| PDCAlpha | 76 | -0,1019 | 0,15520 | 0,01780 | -5,723 | 75 | 0,000 |
| PDCBeta | 76 | -0,0645 | 0,11776 | 0,01351 | -4,777 | 75 | 0,000 |
| PDCGamma | 76 | -0,0359 | 0,11445 | 0,01313 | -2,737 | 75 | 0,008 |
| **Group 12** |  |  |  |  |  |  |  |
| PDCDelta | 76 | 0,0647 | 0,16529 | 0,01896 | 3,415 | 75 | 0,001 |
| PDCTheta | 76 | 0,0894 | 0,15733 | 0,01805 | 4,955 | 75 | 0,000 |
| PDCAlpha | 76 | 0,1019 | 0,15520 | 0,01780 | 5,723 | 75 | 0,000 |
| PDCBeta | 76 | 0,0645 | 0,11776 | 0,01351 | 4,777 | 75 | 0,000 |
| PDCGamma | 76 | 0,0359 | 0,11445 | 0,01313 | 2,737 | 75 | 0,008 |

**Directed transfer function**

| **Variable** | **N** | **mean** | **Std. Deviation** | **Std. errorr mean** | **t** | **df** | **Sign.** |
| --- | --- | --- | --- | --- | --- | --- | --- |
| **Group 1** |  |  |  |  |  |  |  |
| DTFDelta | 115 | -0,1214 | 0,20904 | 0,01949 | -6,226 | 114 | 0,000 |
| DTFTheta | 115 | -0,0960 | 0,25077 | 0,02338 | -4,103 | 114 | 0,000 |
| DTFAlpha | 115 | -0,0533 | 0,22866 | 0,02132 | -2,500 | 114 | 0,014 |
| DTFBeta | 115 | -0,0699 | 0,17227 | 0,01606 | -4,350 | 114 | 0,000 |
| DTFGamma | 115 | -0,0649 | 0,14350 | 0,01338 | -4,848 | 114 | 0,000 |
| **Group 2** |  |  |  |  |  |  |  |
| DTFDelta | 115 | 0,1214 | 0,20904 | 0,01949 | 6,226 | 114 | 0,000 |
| DTFTheta | 115 | 0,0960 | 0,25077 | 0,02338 | 4,103 | 114 | 0,000 |
| DTFAlpha | 115 | 0,0533 | 0,22866 | 0,02132 | 2,500 | 114 | 0,014 |
| DTFBeta | 115 | 0,0699 | 0,17227 | 0,01606 | 4,350 | 114 | 0,000 |
| DTFGamma | 115 | 0,0649 | 0,14350 | 0,01338 | 4,848 | 114 | 0,000 |
| **Group 3** |  |  |  |  |  |  |  |
| DTFDelta | 113 | 0,1284 | 0,13498 | 0,01270 | 10,110 | 112 | 0,000 |
| DTFTheta | 113 | 0,1719 | 0,14956 | 0,01407 | 12,217 | 112 | 0,000 |
| DTFAlpha | 113 | 0,1346 | 0,12556 | 0,01181 | 11,396 | 112 | 0,000 |
| DTFBeta | 113 | 0,0837 | 0,09102 | 0,00856 | 9,777 | 112 | 0,000 |
| DTFGamma | 113 | 0,0542 | 0,06814 | 0,00641 | 8,448 | 112 | 0,000 |
| **Group 4** |  |  |  |  |  |  |  |
| DTFDelta | 113 | -0,1284 | 0,13498 | 0,01270 | -10,110 | 112 | 0,000 |
| DTFTheta | 113 | -0,1719 | 0,14956 | 0,01407 | -12,217 | 112 | 0,000 |
| DTFAlpha | 113 | -0,1346 | 0,12556 | 0,01181 | -11,396 | 112 | 0,000 |
| DTFBeta | 113 | -0,0837 | 0,09102 | 0,00856 | -9,777 | 112 | 0,000 |
| DTFGamma | 113 | -0,0542 | 0,06814 | 0,00641 | -8,448 | 112 | 0,000 |
| **Group 5** |  |  |  |  |  |  |  |
| DTFDelta | 92 | 0,0711 | 0,13226 | 0,01379 | 5,158 | 91 | 0,000 |
| DTFTheta | 92 | 0,0545 | 0,09633 | 0,01004 | 5,425 | 91 | 0,000 |
| DTFAlpha | 92 | 0,0342 | 0,08720 | 0,00909 | 3,760 | 91 | 0,000 |
| DTFBeta | 92 | 0,0281 | 0,06281 | 0,00655 | 4,298 | 91 | 0,000 |
| DTFGamma | 92 | 0,0247 | 0,05878 | 0,00613 | 4,036 | 91 | 0,000 |
| **Group 6** |  |  |  |  |  |  |  |
| DTFDelta | 92 | -0,0711 | 0,13226 | 0,01379 | -5,158 | 91 | 0,000 |
| DTFTheta | 92 | -0,0545 | 0,09633 | 0,01004 | -5,425 | 91 | 0,000 |
| DTFAlpha | 92 | -0,0342 | 0,08720 | 0,00909 | -3,760 | 91 | 0,000 |
| DTFBeta | 92 | -0,0281 | 0,06281 | 0,00655 | -4,298 | 91 | 0,000 |
| DTFGamma | 92 | -0,0247 | 0,05878 | 0,00613 | -4,036 | 91 | 0,000 |
| **Group 7** |  |  |  |  |  |  |  |
| DTFDelta | 92 | 0,1806 | 0,20979 | 0,02187 | 8,259 | 91 | 0,000 |
| DTFTheta | 92 | 0,1764 | 0,22628 | 0,02359 | 7,479 | 91 | 0,000 |
| DTFAlpha | 92 | 0,1445 | 0,23225 | 0,02421 | 5,967 | 91 | 0,000 |
| DTFBeta | 92 | 0,1033 | 0,19809 | 0,02065 | 5,003 | 91 | 0,000 |
| DTFGamma | 92 | 0,0966 | 0,14333 | 0,01494 | 6,465 | 91 | 0,000 |
| **Group 8** |  |  |  |  |  |  |  |
| DTFDelta | 92 | -0,1806 | 0,20979 | 0,02187 | -8,259 | 91 | 0,000 |
| DTFTheta | 92 | -0,1764 | 0,22628 | 0,02359 | -7,479 | 91 | 0,000 |
| DTFAlpha | 92 | -0,1445 | 0,23225 | 0,02421 | -5,967 | 91 | 0,000 |
| DTFBeta | 92 | -0,1033 | 0,19809 | 0,02065 | -5,003 | 91 | 0,000 |
| DTFGamma | 92 | -0,0966 | 0,14333 | 0,01494 | -6,465 | 91 | 0,000 |
| **Group 9** |  |  |  |  |  |  |  |
| DTFDelta | 78 | 0,1182 | 0,14795 | 0,01675 | 7,059 | 77 | 0,000 |
| DTFTheta | 78 | 0,0999 | 0,15004 | 0,01699 | 5,883 | 77 | 0,000 |
| DTFAlpha | 78 | 0,0550 | 0,12438 | 0,01408 | 3,905 | 77 | 0,000 |
| DTFBeta | 78 | 0,0569 | 0,10040 | 0,01137 | 5,009 | 77 | 0,000 |
| DTFGamma | 78 | 0,0636 | 0,10002 | 0,01132 | 5,615 | 77 | 0,000 |
| **Group 10** |  |  |  |  |  |  |  |
| DTFDelta | 78 | -0,1182 | 0,14795 | 0,01675 | -7,059 | 77 | 0,000 |
| DTFTheta | 78 | -0,0999 | 0,15004 | 0,01699 | -5,883 | 77 | 0,000 |
| DTFAlpha | 78 | -0,0550 | 0,12438 | 0,01408 | -3,905 | 77 | 0,000 |
| DTFBeta | 78 | -0,0569 | 0,10040 | 0,01137 | -5,009 | 77 | 0,000 |
| DTFGamma | 78 | -0,0636 | 0,10002 | 0,01132 | -5,615 | 77 | 0,000 |
| **Group 11** |  |  |  |  |  |  |  |
| DTFDelta | 76 | -0,0530 | 0,14129 | 0,01621 | -3,273 | 75 | 0,002 |
| DTFTheta | 76 | -0,0591 | 0,11995 | 0,01376 | -4,297 | 75 | 0,000 |
| DTFAlpha | 76 | -0,0742 | 0,12654 | 0,01451 | -5,115 | 75 | 0,000 |
| DTFBeta | 76 | -0,0553 | 0,10867 | 0,01247 | -4,440 | 75 | 0,000 |
| DTFGamma | 76 | -0,0276 | 0,10714 | 0,01229 | -2,246 | 75 | 0,028 |
| **Group 12** |  |  |  |  |  |  |  |
| DTFDelta | 76 | 0,0530 | 0,14129 | 0,01621 | 3,273 | 75 | 0,002 |
| DTFTheta | 76 | 0,0591 | 0,11995 | 0,01376 | 4,297 | 75 | 0,000 |
| DTFAlpha | 76 | 0,0742 | 0,12654 | 0,01451 | 5,115 | 75 | 0,000 |
| DTFBeta | 76 | 0,0553 | 0,10867 | 0,01247 | 4,440 | 75 | 0,000 |
| DTFGamma | 76 | 0,0276 | 0,10714 | 0,01229 | 2,246 | 75 | 0,028 |

**Granger causality**

| **Variable** | **N** | **mean** | **Std. Deviation** | **Std. errorr mean** | **t** | **df** | **Sign.** |
| --- | --- | --- | --- | --- | --- | --- | --- |
| **Group 1** |  |  |  |  |  |  |  |
| GCDelta | 115 | 0,0006 | 0,08794 | 0,00820 | 0,068 | 114 | 0,946 |
| GCTheta | 115 | -0,0124 | 0,08629 | 0,00805 | -1,543 | 114 | 0,126 |
| GCAlpha | 115 | -0,0156 | 0,09382 | 0,00875 | -1,787 | 114 | 0,077 |
| GCBeta | 115 | -0,0254 | 0,09220 | 0,00860 | -2,956 | 114 | 0,004 |
| GCGamma | 115 | -0,0233 | 0,06264 | 0,00584 | -3,981 | 114 | 0,000 |
| **Group 2** |  |  |  |  |  |  |  |
| GCDelta | 115 | -0,0006 | 0,08794 | 0,00820 | -0,068 | 114 | 0,946 |
| GCTheta | 115 | 0,0124 | 0,08629 | 0,00805 | 1,543 | 114 | 0,126 |
| GCAlpha | 115 | 0,0156 | 0,09382 | 0,00875 | 1,787 | 114 | 0,077 |
| GCBeta | 115 | 0,0254 | 0,09220 | 0,00860 | 2,956 | 114 | 0,004 |
| GCGamma | 115 | 0,0233 | 0,06264 | 0,00584 | 3,981 | 114 | 0,000 |
| **Group 3** |  |  |  |  |  |  |  |
| GCDelta | 113 | 0,0031 | 0,04875 | 0,00459 | 0,667 | 112 | 0,506 |
| GCTheta | 113 | 0,0109 | 0,04778 | 0,00449 | 2,419 | 112 | 0,017 |
| GCAlpha | 113 | 0,0169 | 0,04851 | 0,00456 | 3,709 | 112 | 0,000 |
| GCBeta | 113 | 0,0081 | 0,04355 | 0,00410 | 1,978 | 112 | 0,050 |
| GCGamma | 113 | 0,0047 | 0,02548 | 0,00240 | 1,946 | 112 | 0,054 |
| **Group 4** |  |  |  |  |  |  |  |
| GCDelta | 113 | -0,0031 | 0,04875 | 0,00459 | -0,667 | 112 | 0,506 |
| GCTheta | 113 | -0,0109 | 0,04778 | 0,00449 | -2,419 | 112 | 0,017 |
| GCAlpha | 113 | -0,0169 | 0,04851 | 0,00456 | -3,709 | 112 | 0,000 |
| GCBeta | 113 | -0,0081 | 0,04355 | 0,00410 | -1,978 | 112 | 0,050 |
| GCGamma | 113 | -0,0047 | 0,02548 | 0,00240 | -1,946 | 112 | 0,054 |
| **Group 5** |  |  |  |  |  |  |  |
| GCDelta | 92 | 0,0057 | 0,06412 | 0,00669 | 0,852 | 91 | 0,396 |
| GCTheta | 92 | -0,0180 | 0,04922 | 0,00513 | -3,501 | 91 | 0,001 |
| GCAlpha | 92 | 0,0101 | 0,04739 | 0,00494 | 2,035 | 91 | 0,045 |
| GCBeta | 92 | 0,0176 | 0,04723 | 0,00492 | 3,573 | 91 | 0,001 |
| GCGamma | 92 | 0,0079 | 0,04826 | 0,00503 | 1,580 | 91 | 0,118 |
| **Group 6** |  |  |  |  |  |  |  |
| GCDelta | 92 | -0,0057 | 0,06412 | 0,00669 | -0,852 | 91 | 0,396 |
| GCTheta | 92 | 0,0180 | 0,04922 | 0,00513 | 3,501 | 91 | 0,001 |
| GCAlpha | 92 | -0,0101 | 0,04739 | 0,00494 | -2,035 | 91 | 0,045 |
| GCBeta | 92 | -0,0176 | 0,04723 | 0,00492 | -3,573 | 91 | 0,001 |
| GCGamma | 92 | -0,0079 | 0,04826 | 0,00503 | -1,580 | 91 | 0,118 |
| **Group 7** |  |  |  |  |  |  |  |
| GCDelta | 92 | -0,0109 | 0,09292 | 0,00969 | -1,124 | 91 | 0,264 |
| GCTheta | 92 | 0,0101 | 0,05476 | 0,00571 | 1,761 | 91 | 0,082 |
| GCAlpha | 92 | 0,0106 | 0,04260 | 0,00444 | 2,379 | 91 | 0,019 |
| GCBeta | 92 | 0,0118 | 0,03441 | 0,00359 | 3,300 | 91 | 0,001 |
| GCGamma | 92 | 0,0146 | 0,03057 | 0,00319 | 4,596 | 91 | 0,000 |
| **Group 8** |  |  |  |  |  |  |  |
| GCDelta | 92 | 0,0109 | 0,09292 | 0,00969 | 1,124 | 91 | 0,264 |
| GCTheta | 92 | -0,0101 | 0,05476 | 0,00571 | -1,761 | 91 | 0,082 |
| GCAlpha | 92 | -0,0106 | 0,04260 | 0,00444 | -2,379 | 91 | 0,019 |
| GCBeta | 92 | -0,0118 | 0,03441 | 0,00359 | -3,300 | 91 | 0,001 |
| GCGamma | 92 | -0,0146 | 0,03057 | 0,00319 | -4,596 | 91 | 0,000 |
| **Group 9** |  |  |  |  |  |  |  |
| GCDelta | 78 | 0,0137 | 0,04794 | 0,00543 | 2,515 | 77 | 0,014 |
| GCTheta | 78 | 0,0270 | 0,04664 | 0,00528 | 5,117 | 77 | 0,000 |
| GCAlpha | 78 | 0,0550 | 0,05070 | 0,00574 | 9,579 | 77 | 0,000 |
| GCBeta | 78 | 0,0634 | 0,06068 | 0,00687 | 9,233 | 77 | 0,000 |
| GCGamma | 78 | 0,0391 | 0,04629 | 0,00524 | 7,460 | 77 | 0,000 |
| **Group 10** |  |  |  |  |  |  |  |
| GCDelta | 78 | -0,0137 | 0,04794 | 0,00543 | -2,515 | 77 | 0,014 |
| GCTheta | 78 | -0,0270 | 0,04664 | 0,00528 | -5,117 | 77 | 0,000 |
| GCAlpha | 78 | -0,0550 | 0,05070 | 0,00574 | -9,579 | 77 | 0,000 |
| GCBeta | 78 | -0,0634 | 0,06068 | 0,00687 | -9,233 | 77 | 0,000 |
| GCGamma | 78 | -0,0391 | 0,04629 | 0,00524 | -7,460 | 77 | 0,000 |
| **Group 11** |  |  |  |  |  |  |  |
| GCDelta | 76 | 0,0040 | 0,04822 | 0,00553 | 0,719 | 75 | 0,475 |
| GCTheta | 76 | 0,0047 | 0,04303 | 0,00494 | 0,956 | 75 | 0,342 |
| GCAlpha | 76 | 0,0094 | 0,04871 | 0,00559 | 1,679 | 75 | 0,097 |
| GCBeta | 76 | 0,0135 | 0,05150 | 0,00591 | 2,287 | 75 | 0,025 |
| GCGamma | 76 | 0,0073 | 0,03929 | 0,00451 | 1,620 | 75 | 0,110 |
| **Group 12** |  |  |  |  |  |  |  |
| GCDelta | 76 | -0,0040 | 0,04822 | 0,00553 | -0,719 | 75 | 0,475 |
| GCTheta | 76 | -0,0047 | 0,04303 | 0,00494 | -0,956 | 75 | 0,342 |
| GCAlpha | 76 | -0,0094 | 0,04871 | 0,00559 | -1,679 | 75 | 0,097 |
| GCBeta | 76 | -0,0135 | 0,05150 | 0,00591 | -2,287 | 75 | 0,025 |
| GCGamma | 76 | -0,0073 | 0,03929 | 0,00451 | -1,620 | 75 | 0,110 |

1. To assess stability of the resting state connectivity measures the epochs were split into two halves representing early and late epochs. Connectivity measures for both halves are presented graphically below.

Supp. Fig. 1a.

Difference in the PDC (Partial Directed Coherence) index demonstrating prevailing directionality in the bidirectional connections among the ROI pairs *in the first 5-min segment*.


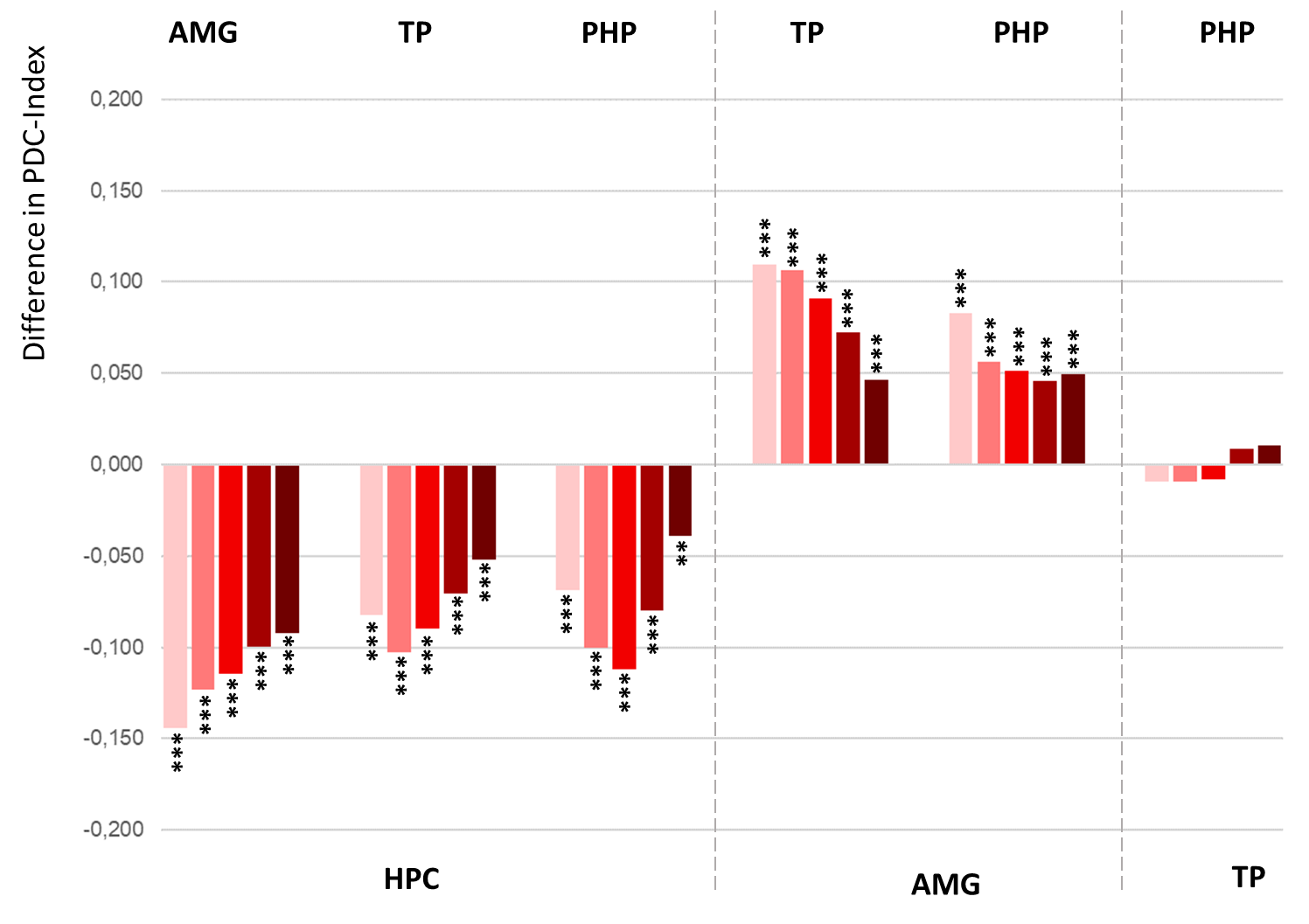


Supp. Fig. 1b.

Difference in the PDC (Partial Directed Coherence) index demonstrating prevailing directionality in the bidirectional connections among the ROI pairs *in the second 5-min segment*.


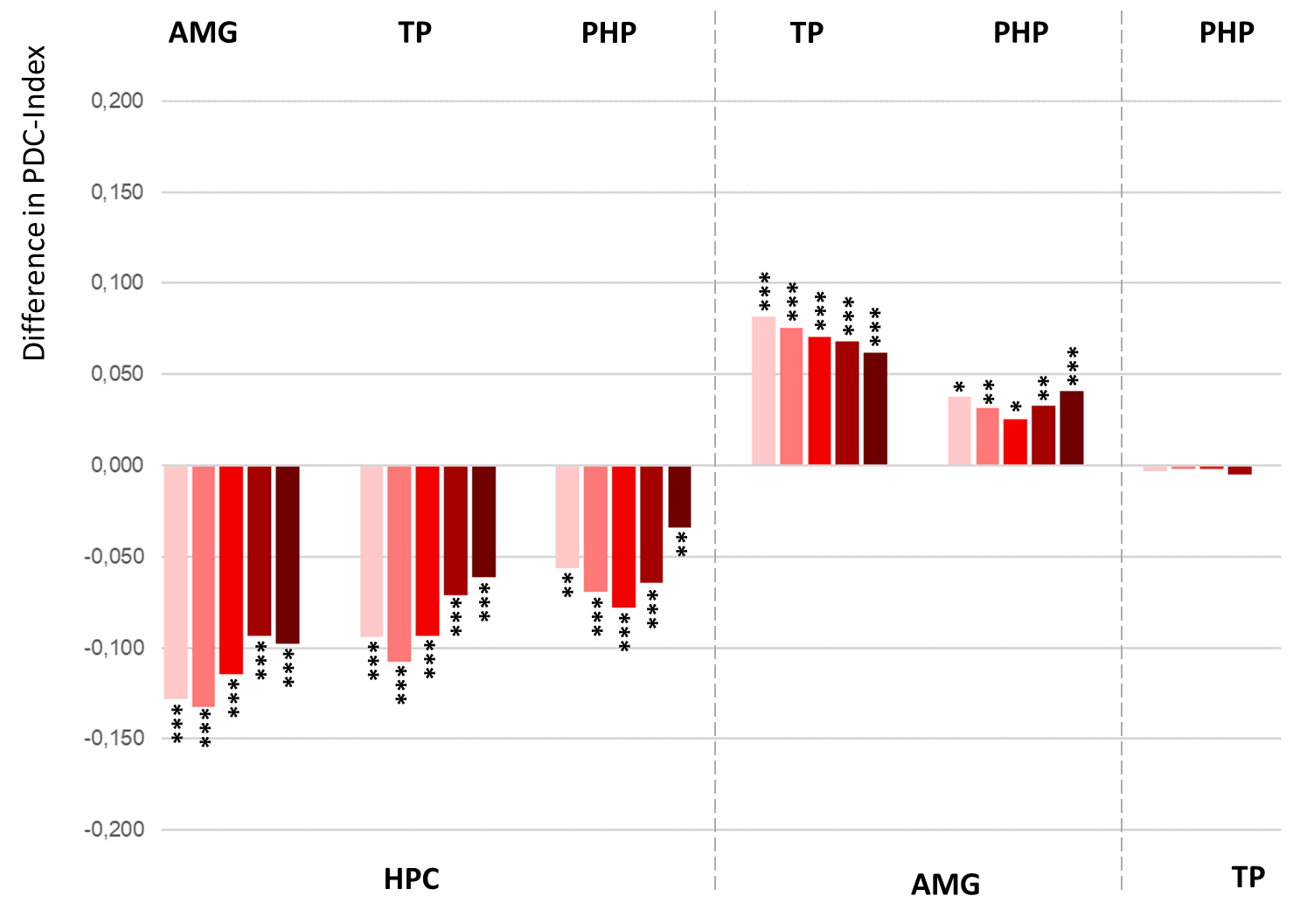


Supp. Fig. 2a.

Difference in the DTF (Directed Transfer Function) index demonstrating prevailing directionality in the bidirectional connections among the ROI pairs *in the first 5-min segment*.


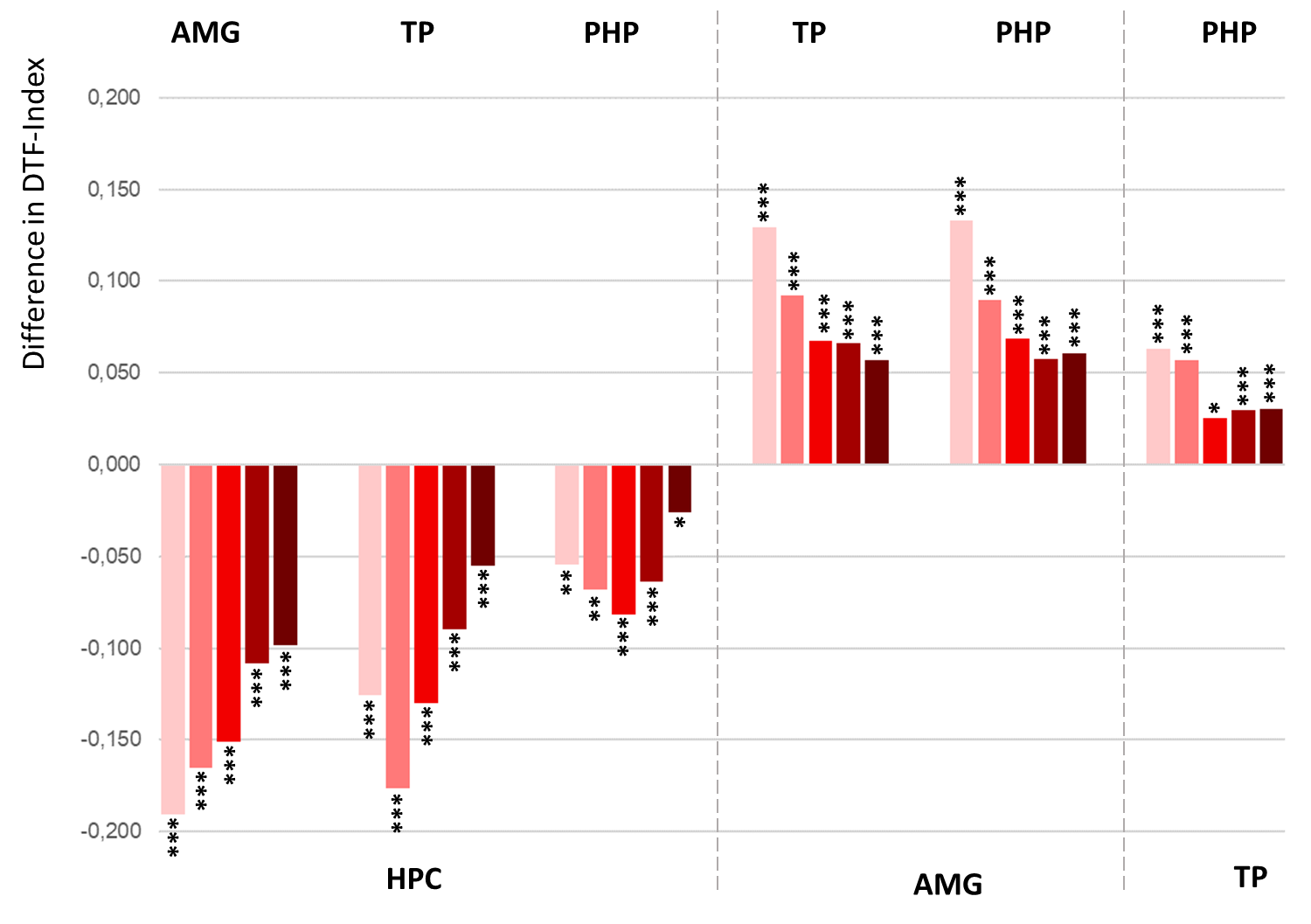


Supp. Fig. 2b.

Difference in the DTF (Directed Transfer Function) index demonstrating prevailing directionality in the bidirectional connections among the ROI pairs *in the second 5-min segment*.


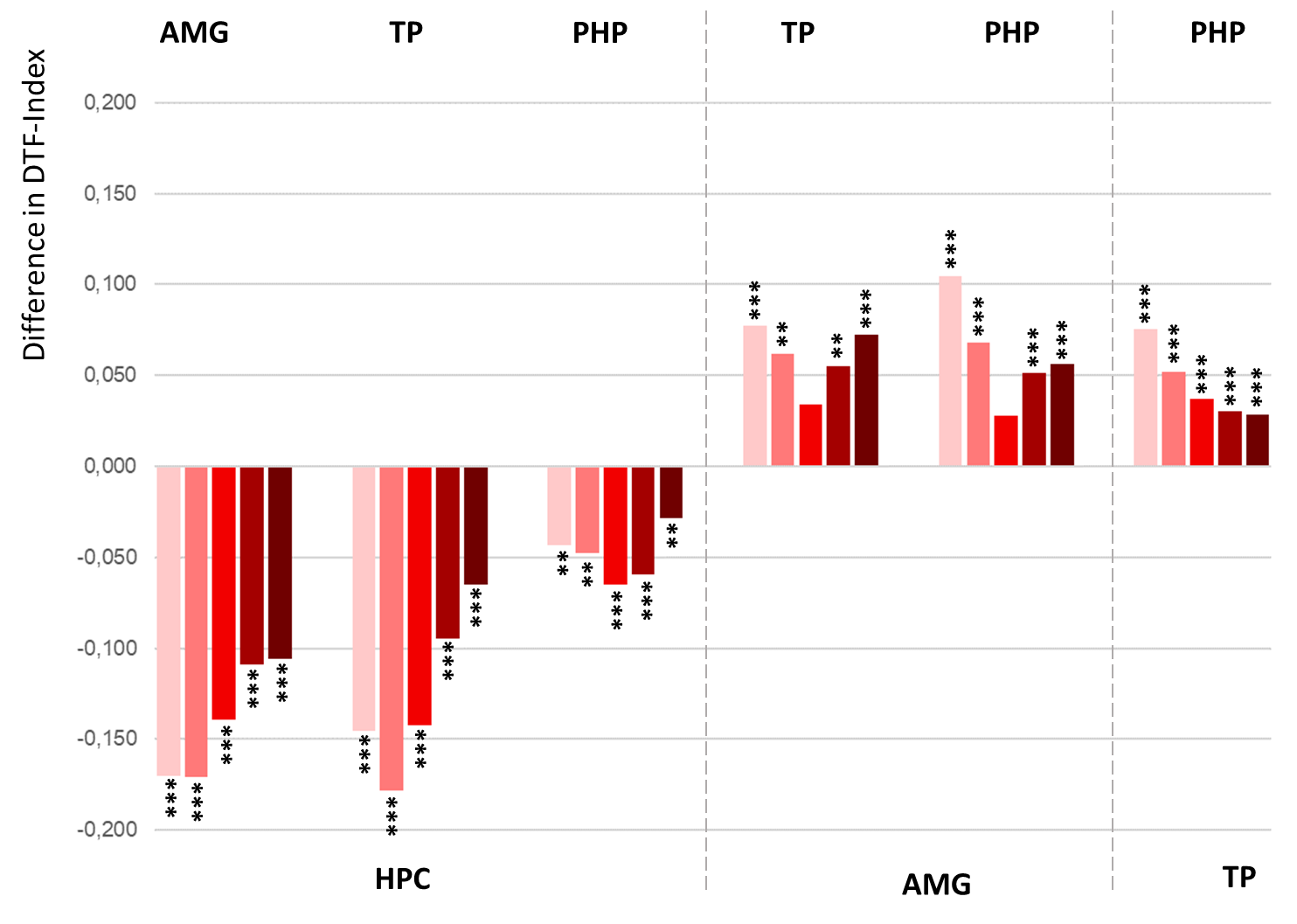


Supp. Fig. 3a.

Difference in the Granger Causality index demonstrating prevailing directionality in the bidirectional connections among the ROI pairs *in the first 5-min segment*.


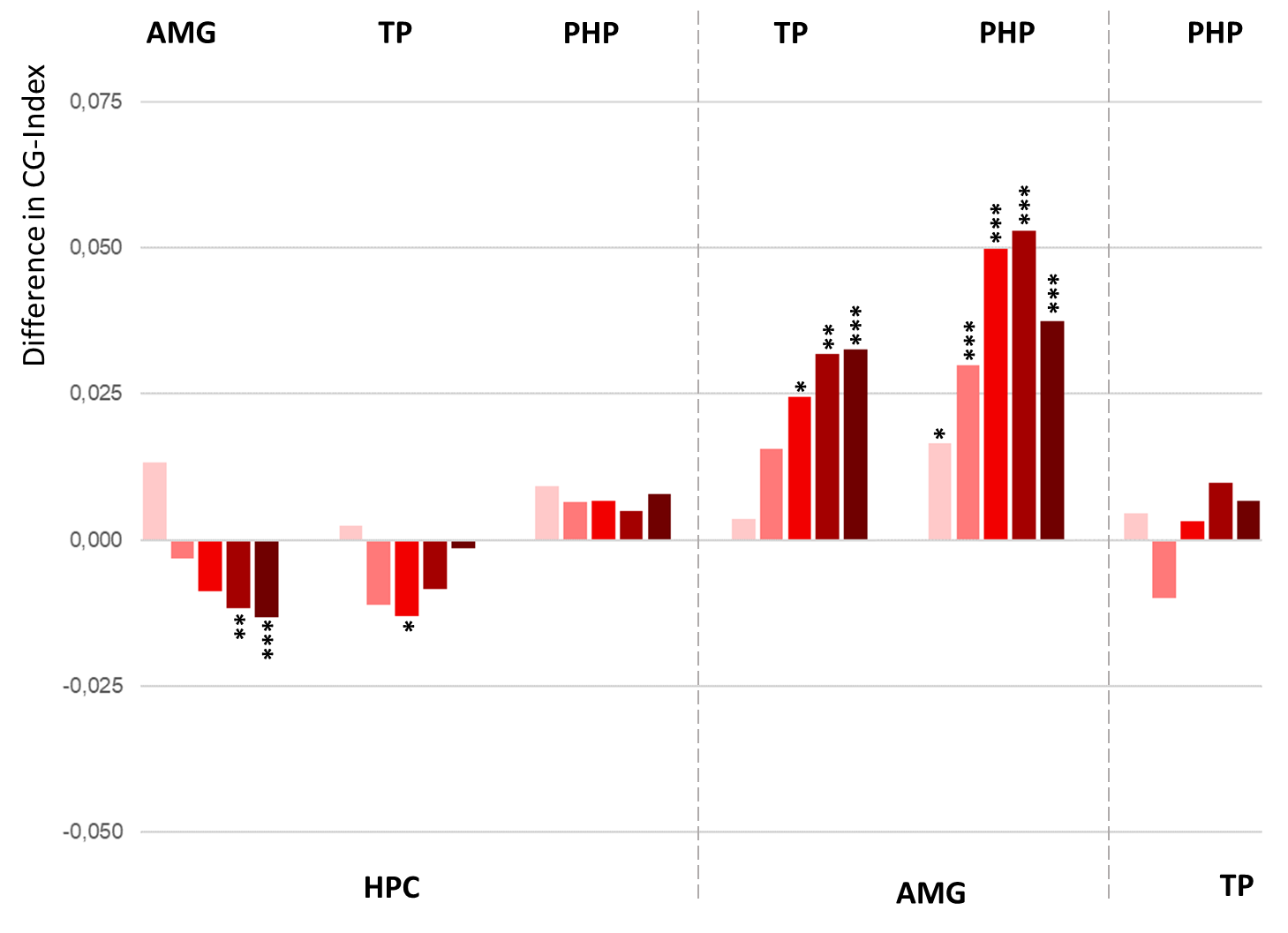


Supp. Fig. 3b.

Difference in the Granger Causality index demonstrating prevailing directionality in the bidirectional connections among the ROI pairs *in the second 5-min segment*.


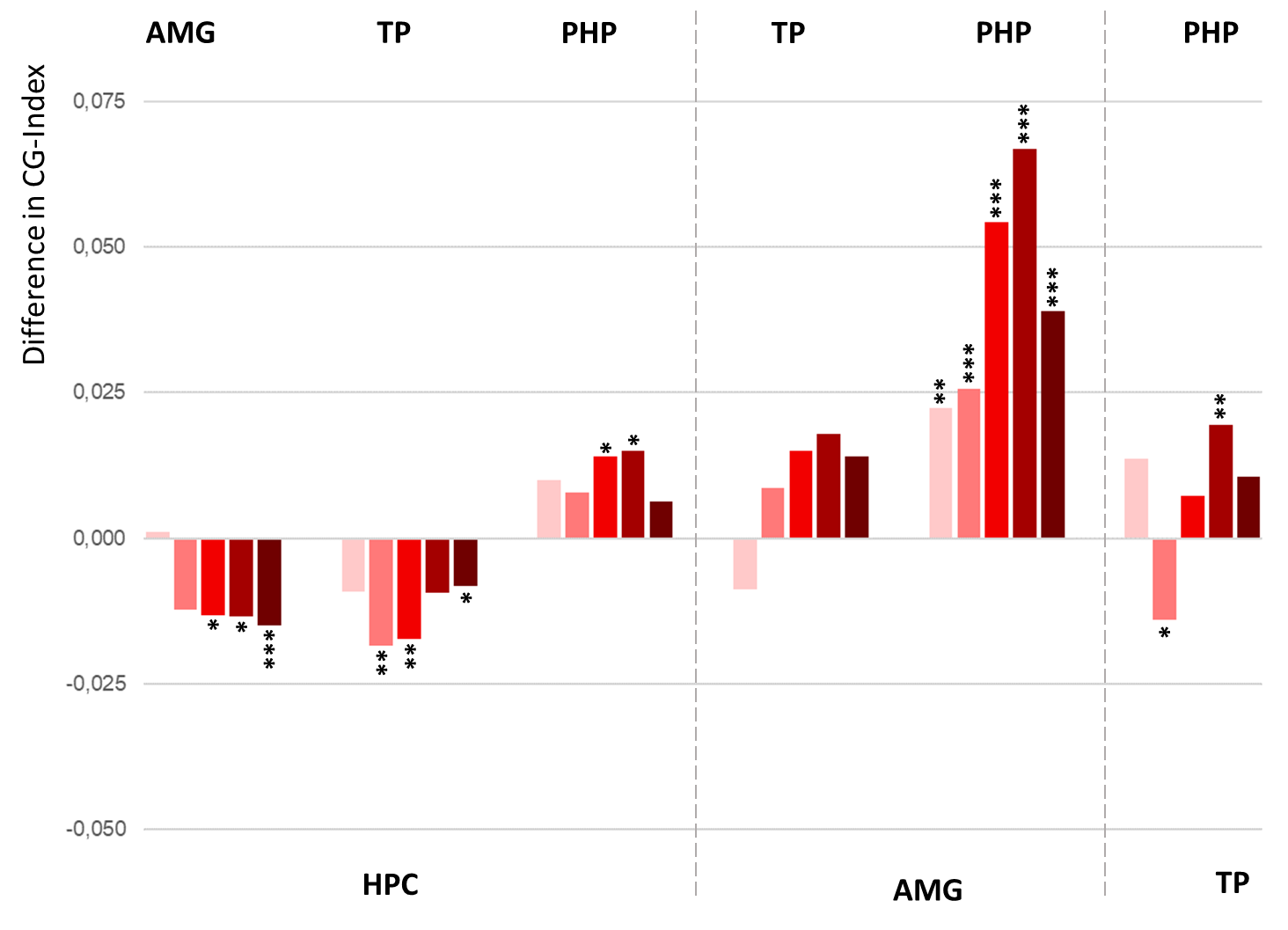


For all plots: Each bunch of bars represents five colour coded frequency bands: delta (pink), theta (light red), alpha (red), beta (dark red) and low gamma (brown), ordered from left to right. The up- or downward orientation of the bars on the zero line indicates the direction of connectivity towards the ROIs assigned above and below the plot. Respectively, the ROIs of signal origin are assigned on the opposite side of the plot. HPC: hippocampus, AMG: amygdala, TP: temporal pole, PHP: parahippocampal gyrus

*** – p < 0.001, ** – p < 0.01, * - p < 0.5

1. Supp. Table 1.

Characteristics of the SPES-evoked potentials.

| STIM site | REC site | N | Amplitude z-score | | Latency to peak, msec | |
| --- | --- | --- | --- | --- | --- | --- |
|  |  |  | Mean | SEM | Mean | SEM |
| AMG | HPC | 39 | 39.9 | 6.9 | 44.6 | 3.3 |
|  | PHP | 29 | 11.3 | 1.8 | 50.1 | 3.9 |
|  | TP | 46 | 28.2 | 5.1 | 47.6 | 4.7 |
| HPC | AMG | 39 | 19.3 | 2.9 | 22.3 | 2.6 |
|  | PHP | 28 | 51.7 | 10.4 | 24.9 | 4.6 |
|  | TP | 47 | 7.0 | 0.8 | 31.9 | 3.6 |
| PHP | AMG | 39 | 9.0 | 1.7 | 48.5 | 5.3 |
|  | HPC | 37 | 14.8 | 1.5 | 41.7 | 4.7 |
|  | TP | 42 | 3.9 | 0.5 | 42.3 | 5.2 |
| TP | AMG | 35 | 19.1 | 2.4 | 34.5 | 3.1 |
|  | HPC | 35 | 23.4 | 3.6 | 48.7 | 3.8 |
|  | PHP | 21 | 15.6 | 1.8 | 46.2 | 2.2 |

Amplitude and latency of N1 peak of the evoked potentials regarding stimulation (STIM) and recording (REC) sites. N represents the number of recording sites for each ROI. HPC: hippocampus, AMG: amygdala, TP: temporal pole, PHP: parahippocampal gyrus
