# Supplementary material for: Intracranial EEG-Based Directed Functional Connectivity in Alpha to Gamma Frequency Range Reflects Local Circuits of the Human Mesiotemporal Network
Source: Brain Topogr. 2024 Oct 22;38(1):10. doi: 10.1007/s10548-024-01084-w (PMC11496326; doi:10.1007/s10548-024-01084-w)
Supplement: Supplementary file 2 — Supplementary file2 (DOCX 28 kb) [file 10548_2024_1084_MOESM2_ESM.docx]

Table 2.

Statistical details obtained for the Granger Causality, Partial Directed Coherence and Directed Transfer Function analysis.

1. F and p values for all ROI pairs and frequency bands

| **ROI pairs** | **delta** | **theta** | **alpha** | **beta** | **low gamma** | **method** |
| --- | --- | --- | --- | --- | --- | --- |
| AMG → HPC | F_1,166_=2.004,  p = 0.16 | F_1,166_=0.93,  p = 0.34 | F_1,166_=2.18,  p = 0.14 | F_1,166_=6.64,  p = 0.011 * | F_1,166_=19.85,  p = 0.000 *** | **Granger Causality** |
| TP → HPC | F_1,218_=0.05,  p = 0.83 | F_1,218_=3.15,  p = 0.08 | F_1,218_=7.61,  p = 0.006 ** | F_1,218_=1.52,  p = 0.22 | F_1,218_=1.5,  p = 0.22 |  |
| PHP → HPC | F_1,144_=0.72,  p = 0.4 | F_1,144_=0.77,  p = 0.38 | F_1,144_=3.20,  p = 0.076 | F_1,144_=4.9,  p = 0.027 * | F_1,144_=2.69,  p = 0.103 |  |
| AMG → TP | F_1,216_=0.22,  p = 0.64 | F_1,216_=1.17,  p = 0.28 | F_1,216_=2.06,  p = 0.15 | F_1,216_=6.22,  p = 0.013 * | F_1,216_=12.10,  p = 0.001 ** |  |
| AMG → PHP | F_1,144_=42.06,  p < 0.000 | F_1,144_=14.49,  p = 0.000 *** | F_1,144_=63.63,  p = 0.000 *** | F_1,144_=59.86,  p = 0.000 *** | F_1,144_=44.52,  p = 0.000 *** |  |
| TP ↔ PHP | F_1,176_=0.39,  p = 0.53 | F_1,176_=9.47,  p = 0.002 ** | F_1,176_=2.23,  p = 0.13 | F_1,176_=8.03,  p = 0.005 ** | F_1,176_=2.95,  p = 0.088 |  |
| AMG → HPC | F_1,166_=49.18,  p = 0.000 *** | F_1,166_=48.63,  p = 0.000 *** | F_1,166_=42.12,  p = 0.000 *** | F_1,166_=33.65,  p = 0.000 *** | F_1,166_=53.70,  p = 0.000 *** | **Partial Directed Coherence** |
| TP → HPC | F_1,218_=28.47,  p = 0.000 *** | F_1,218_=55.26,  p = 0.000 *** | F_1,218_=46.87,  p = 0.000 *** | F_1,218_=40.04,  p = 0.000 *** | F_1,218_=53.84,  p = 0.000 *** |  |
| PHP → HPC | F_1,144_=14.23,  p = 0.000 *** | F_1,144_=26.78,  p = 0.000 *** | F_1,144_=36.48,  p = 0.000 *** | F_1,144_=24.37,  p = 0.000 *** | F_1,144_=9.86,  p = 0.002 ** |  |
| AMG → TP | F_1,216_=63.57,  p = 0.000 *** | F_1,216_=63.93,  p = 0.000 *** | F_1,216_=47.61,  p = 0.000 *** | F_1,216_=38.87,  p = 0.000 *** | F_1,216_=26.30,  p = 0.000 *** |  |
| AMG → PHP | F_1,144_=14.19,  p = 0.000 *** | F_1,144_=25.94,  p = 0.000 *** | F_1,144_=15.41,  p = 0.000 *** | F_1,144_=26.47,  p = 0.000 *** | F_1,144_=36.28,  p = 0.000 *** |  |
| TP – PHP | F_1,176_=0.07,  p = 0.79 | F_1,176_=0.67,  p = 0.41 | F_1,176_=0.011,  p = 0.92 | F_1,176_=0.69,  p = 0.41 | F_1,176_=1.98,  p = 0.16 |  |
| AMG → HPC | F_1,166_=77.97,  p = 0.000 *** | F_1,166_=68.81,  p = 0.000 *** | F_1,166_=43.98,  p = 0.000 *** | F_1,166_=36.38,  p = 0.000 *** | F_1,166_=52.20,  p = 0.000 *** | **Directed Transfer Function** |
| TP → HPC | F_1,218_=88.86,  p = 0.000 *** | F_1,218_=149.83,  p = 0.000 *** | F_1,218_=129.64,  p = 0.000 *** | F_1,218_=85.07,  p = 0.000 *** | F_1,218_=74.48,  p = 0.000 *** |  |
| PHP → HPC | F_1,144_=11.91,  p = 0.001 ** | F_1,144_=20.87,  p = 0.000 *** | F_1,144_=32.1,  p = 0.000 *** | F_1,144_=21.49,  p = 0.000 *** | F_1,144_=6.54,  p = 0.012 * |  |
| AMG → TP | F_1,216_=35.19,  p = 0.000 *** | F_1,216_=25.06,  p = 0.000 *** | F_1,216_=7.77,  p = 0.006 ** | F_1,216_=19.63,  p = 0.000 *** | F_1,216_=22.76,  p = 0.000 *** |  |
| AMG → PHP | F_1,144_=42.06,  p = 0.000 *** | F_1,144_=31.75,  p = 0.000 *** | F_1,144_=11.23,  p = 0.001 ** | F_1,144_=27.55,  p = 0.000 *** | F_1,144_=34.15,  p = 0.000 *** |  |
| TP → PHP | F_1,176_=28.85,  p = 0.000 *** | F_1,176_=28.21,  p = 0.000 *** | F_1,176_=13.85,  p = 0.000 *** | F_1,176_=21.20,  p = 0.000 *** | F_1,176_=24.13,  p = 0.000 *** |  |

1. F and p values for all ROI pairs and frequency bands after exclusion of the recording contacts placed in seizure onset zone

| **ROI pairs** | **delta** | **theta** | **alpha** | **beta** | **low gamma** | **method** |
| --- | --- | --- | --- | --- | --- | --- |
| AMG → HPC | F_1,96_=1.14,  p = 0.29 | F_1,96_=0.021,  p = 0.89 | F_1,96_=0.36,  p = 0.55 | F_1,96_=8.19,  p = 0.005 ** | F_1,96_=16.11,  p = 0.000 *** | **Granger Causality** |
| TP → HPC | F_1,120_=0.41,  p = 0.53 | F_1,120_=7.26,  p = 0.008 ** | F_1,120_=1.09,  p = 0.300 | F_1,120_=1.55,  p = 0.215 | F_1,120_=2.05,  p = 0.155 |  |
| PHP → HPC | F_1,80_=1.13,  p = 0.29 | F_1,80_=1.15,  p = 0.23 | F_1,80_=1.15,  p = 0.23 | F_1,80_=1.54,  p = 0.22 | F_1,80_=3.42,  p = 0.068 |  |
| AMG → TP | F_1,186_=0.005,  p = 0.94 | F_1,186_=0.133,  p = 0.72 | F_1,186_=0.11,  p = 0.74 | F_1,186_=0.65,  p = 0.200 | F_1,186_=2.98,  p = 0.086 |  |
| AMG → PHP | F_1,144_=3.84,  p = 0.052 | F_1,144_=14.45,  p = 0.000 *** | F_1,144_=63.63,  p = 0.000 *** | F_1,144_=59.86,  p = 0.000 *** | F_1,144_=44.52,  p = 0.000 *** |  |
| TP ↔ PHP | F_1,146_=0.36,  p = 0.55 | F_1,146_=6.48,  p = 0.012 * | F_1,146_=4.24,  p = 0.041 * | F_1,146_=11.18,  p = 0.001 ** | F_1,146_=7.22,  p = 0.008 ** |  |
| AMG → HPC | F_1,96_=21.92,  p = 0.000 *** | F_1,96_=21.64,  p = 0.000 *** | F_1,96_=22.04,  p = 0.000 *** | F_1,96_=26.50,  p = 0.000 *** | F_1,96_=36.49,  p = 0.000 *** | **Partial Directed Coherence** |
| TP → HPC | F_1,120_=24.72,  p = 0.000 *** | F_1,120_=49.72,  p = 0.000 *** | F_1,120_=35.01,  p = 0.000 *** | F_1,120_=35.94,  p = 0.000 *** | F_1,120_=38.75,  p = 0.000 *** |  |
| PHP → HPC | F_1,80_=5.84,  p = 0.018 * | F_1,80_=9.24,  p = 0.003 ** | F_1,80_=14.31,  p = 0.000 *** | F_1,80_=8.19,  p = 0.005 ** | F_1,80_=1.94,  p = 0.167 |  |
| AMG → TP | F_1,186_=45.00,  p = 0.000 *** | F_1,186_=46.47,  p = 0.000 *** | F_1,186_=33.96,  p = 0.000 *** | F_1,186_=26.11,  p = 0.000 *** | F_1,186_=14.39,  p = 0.000 *** |  |
| AMG → PHP | F_1,144_=14.19,  p = 0.000 *** | F_1,144_=25.94,  p = 0.000 *** | F_1,144_=15.41,  p = 0.000 *** | F_1,144_=26.47,  p = 0.000 *** | F_1,144_=36.28,  p = 0.000 *** |  |
| TP – PHP | F_1,146_=2.41,  p = 0.12 | F_1,146_=1.30,  p = 0.26 | F_1,146_=3.48,  p = 0.064 | F_1,146_=2.78,  p = 0.098 | F_1,146_=6.73,  p = 0.0104 * |  |
| AMG → HPC | F_1,96_=32.30,  p = 0.000 *** | F_1,96_=26.86,  p = 0.000 *** | F_1,96_=19.78,  p = 0.000 *** | F_1,96_=23.53,  p = 0.000 *** | F_1,96_=34.40,  p = 0.000 *** | **Directed Transfer Function** |
| TP → HPC | F_1,120_=70.48,  p = 0.000 *** | F_1,120_=77.48,  p = 0.000 *** | F_1,120_=58.64,  p = 0.000 *** | F_1,120_=50.94,  p = 0.000 *** | F_1,120_=44.04,  p = 0.000 *** |  |
| PHP → HPC | F_1,80_=5.83,  p = 0.018 * | F_1,80_=7.47,  p = 0.008 ** | F_1,80_=11.45,  p = 0.001 ** | F_1,80_=6.71,  p = 0.011 * | F_1,80_=1.41,  p = 0.238 |  |
| AMG → TP | F_1,186_=24.01,  p = 0.000 *** | F_1,186_=11.51,  p = 0.001 ** | F_1,186_=2.34,  p = 0.128 | F_1,186_=11.29,  p = 0.001 ** | F_1,186_=13.68,  p = 0.000 *** |  |
| AMG → PHP | F_1,144_=42.06,  p = 0.000 *** | F_1,144_=31.75,  p = 0.000 *** | F_1,144_=11.30,  p = 0.001 ** | F_1,144_=27.55,  p = 0.000 *** | F_1,144_=34.12,  p = 0.000 *** |  |
| TP → PHP | F_1,146_=44.17,  p = 0.000 *** | F_1,146_=45.16,  p = 0.000 *** | F_1,146_=33.38,  p = 0.000 *** | F_1,146_=35.33,  p = 0.000 *** | F_1,146_=36.41,  p = 0.000 *** |  |

AMG: amygdala, HPC: hippocampus, TP: temporal pole, PHP: parahippocampal gyrus
